# Quasi‐Continuous Efficient Regulation of Single‐Molecule Electronic Distribution

**DOI:** 10.1002/advs.202412260

**Published:** 2025-02-19

**Authors:** Zhizhou Li, Weilin Hu, Shuyao Zhou, Changqing Xu, Mingyao Li, Jinying Wang, Suhang He, Liang Zhang, Chuancheng Jia, Xuefeng Guo

**Affiliations:** ^1^ Beijing National Laboratory for Molecular Sciences National Biomedical Imaging Center College of Chemistry and Molecular Engineering Peking University 292 Chengfu Road Haidian District Beijing 100871 P. R. China; ^2^ School of Chemistry and Molecular Engineering East China Normal University 3663 North Zhongshan Road, Putuo District Shanghai 200062 P. R. China; ^3^ Center of Single‐Molecule Sciences Institute of Modern Optics Frontiers Science Center for New Organic Matter Tianjin Key Laboratory of Micro‐scale Optical Information Science and Technology College of Electronic Information and Optical Engineering Nankai University 38 Tongyan Road Jinnan District Tianjin 300350 P. R. China

**Keywords:** crown ether, molecular dipole, single‐molecule electronics, through‐space interaction

## Abstract

The electronic distribution characteristics of molecules significantly influence the charge transport properties and the device performance of molecular electronic devices. These characteristics are closely related to subtle molecular structures, forming a formidable challenge for effective control. Here, a flexible crown ether moiety is integrated into the single‐molecule junction, where its spatial structure can be regulated by an external electric field, enabling efficient tuning of the electronic characteristics. It is found that the transformation barriers between different structures and the conductance of single‐molecule junctions can be adjusted by the external electric field. Both theoretical and experimental results consistently demonstrate that the interaction between the external electric field and the intrinsic molecular dipole can alter the molecular energy and stabilize a series of metastable molecular configurations, allowing for the quasi‐continuous manipulation of the electronic characteristics. This study unveils efficient control of the single‐molecule electronic characteristics by external electric fields, advancing a deeper understanding of molecular electronics and supramolecular chemistry.

## Graphical abstract

Through molecular engineering, a crown ether is covalently integrated into a single‐molecule junction to investigate the quasi‐continuous manipulation of the electronic characteristics of molecules. It is found that the internal asymmetric molecular dipole of the flexible crown ether plays an important role in the regulation of electric signals and orbital alignments.

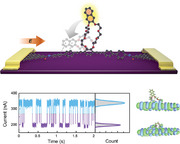

## 1 Introduction

Single‐molecule electronic devices stand as strong candidates to achieve the utmost limits of chip miniaturization, concurrently providing an effective platform for the in‐depth exploration of intrinsic principles at the single‐molecule level.^[^ ^1^, ^2^, ^3^, ^4^, ^5^ ^]^ In particular, single‐molecule analysis provides the capacity to discern the subtle structural variations within molecules,^[^ ^6^ ^]^ which are in general obscured by the averaging effects in ensemble experiments.^[^ ^7^, ^8^ ^]^ By carefully adjusting the molecular structure, its electronic properties can be modulated accordingly.^[^ ^9^, ^10^ ^]^ These changes exert significant influence on charge transport characteristics of single‐molecule electronic devices, possibly engendering remarkable phenomena, such as negative differential conductance,^[^ ^11^ ^]^ and quantum interference.^[^ ^12^ ^]^ Previous reports have proven that the electronic character of a single molecule can be fine‐tuned through strategies such as mechanical extension,^[^ ^9^ ^]^ light stimulation,^[^ ^13^ ^]^ and local chemical reaction.^[^ ^14^ ^]^ As a result, single‐molecule junctions usually generate reversible transitions among different configurations, electronic states, spin configurations and so on.^[^ ^15^, ^16^ ^]^ Nonetheless, efficient modulation of single‐molecule structures through external electric fields remains challenging.

Artificial macrocyclic molecules have been extensively used as host molecules in molecular recognition.^[^ ^17^, ^18^, ^19^, ^20^ ^]^ For instance, crown ethers possess specific selectivity toward metal ions and small organic molecules, which can be used to design and construct molecular machines such as molecular muscles and shuttles.^[^ ^21^ ^]^ Macrocyclic compounds are crucial for constructing stable supramolecular structures, but they are also susceptible to subtle changes in the external environments.^[^ ^22^, ^23^ ^]^ With macroscopic monitoring methods such as nuclear magnetic resonance, circular dichroism spectra, optical emission and absorption spectra, previous researches focused on the behavior of ensemble molecules or small amounts of molecules. In macroscopic experiments, this dynamic microstructure of crown ether is often obscured by the ensemble average effect, making it difficult to accurately characterize using commonly used techniques. However, these microstructural dynamics of flexible crown ethers at the single‐molecule level become critical for the successful assembly and preset operation of molecular machines. Therefore, it is important to monitor these subtle molecular structures and their corresponding changes at the single‐molecule level which still poses a significant hurdle.^[^ ^24^, ^25^, ^26^ ^]^

**Figure 1:**
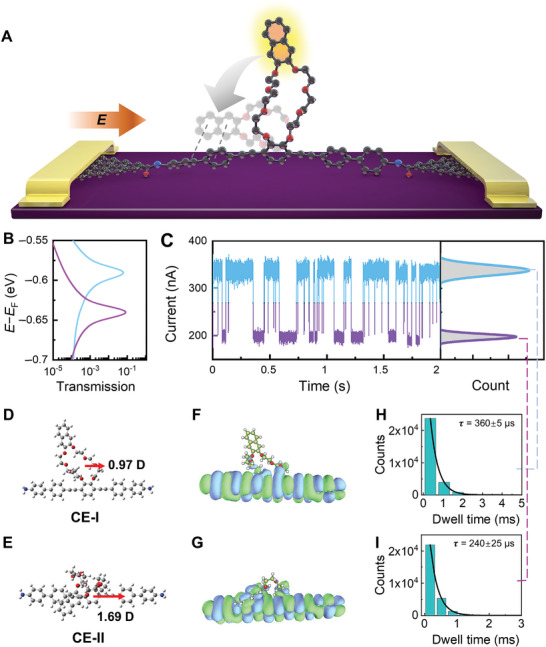
Basic characteristics of crown ether‐based GMG‐SMJs. A) Schematic representation of the device with a single CE molecule. B) Transmission spectra of CE‐I (blue line) and CE‐II (purple line), where *p‐*HOMOs are displayed. C) Left panel: Representative *I−t* trajectory of a single‐molecule junction at 150 K under 0.05 V; Right panel: Corresponding histograms of the conductance states. D,E) Optimized molecular structures of CE‐I and CE‐II, where the molecular dipole components in the direction of the electric field are marked. F,G) Distributions of corresponding HOMOs. H,I) Plots of time intervals of corresponding conductance states.

Building upon the graphene‐molecule‐graphene single‐molecule junction (GMG‐SMJ),^[^ ^27^ ^]^ a single 1,4‐bis(phenylethynyl)benzene molecule, chemically modified with a 24‐crown‐8 ether (CE) side group, is covalently anchored to the carboxyl groups at the edges of graphene electrodes, forming a stable connection through amide groups (**Figure** **1**A). This establishes the foundation for robust testing of long‐term current fluctuations that can reflect the chemical and structural changes in a single molecule. The CE moiety has a flexible ring structure that can self‐adjust in response to the external environment, e.g. electric fields.^[^ ^28^, ^29^ ^]^ This opens the opportunity to investigate the field effect on a molecule, by modulating its electronic structural characteristics.

## 2 Results and Discussion

### 2.1 Basic Characteristics of Crown Ether‐Based GMG‐SMJs

Single‐layer graphene was prepared on the copper foil by chemical vapor deposition and then transferred to the silicon wafer. Continuous lithography and thermal evaporation operations were conducted to deposit the gold electrode on the silicon wafer, forming the graphene transistor with metal‐graphene‐metal contact. Next, polymethyl methacrylate (PMMA) was spin‐coated onto the device, and the electron‐sensitive PMMA on the strips was exposed by dashed line etching^[^ ^27^ ^]^ and electron beam lithography to open the nanoscale window array. After oxygen plasma etching, graphene at the window would be removed, and the carboxyl group would be introduced to the graphene edge at the same time. As the etching time increased, the window would be further extended. Through real‐time current monitoring, the moment when the current dropped to fA‐pA scale can be monitored, and graphene between the electrodes can be considered as disconnected. The spacing between the electrodes would probably be in the vicinity of the nm range. With amino terminals, the molecule of nm‐level length can be connected between the carboxyl‐modified graphene electrode pairs. In the process of connecting molecules, molecular concentration in the reaction solution was very low (≈0.1 mg·mL^−1^). After connection, the device was washed by solvent, to ensure the connection of a single molecule to a certain extent, rather than the molecular packing. The bridging molecule, featuring a crown ether functional center and amino terminals, was synthesized and covalently integrated into graphene electrodes to form stable GMG‐SMJ (Figure 1A).^[^ ^27^ ^]^ The synthesis methods (Schemes S1−S3, Supporting Information), structure characterizations (Figures S1−S8, Supporting Information) and details of the device fabrication (Figures S9−S11, Supporting Information) have been exhibited in the Supporting Information. Before connecting the molecules, the electrodes were disconnected due to the etching of oxygen plasma, and current–voltage (*I*−*V*) curve dropped down to zero. After connecting the molecule between source and drain electrodes, the current signal recovered to some extent. The successful fabrication of GMG‐SMJs was assessed by comparing the *I*−*V* curves obtained before (down to zero) and after (recovered to some extent) connecting the molecule between source and drain electrodes (Figure S12, Supporting Information). Under optimized conditions, the connection yield reached ≈11%, and ≈8 out of 73 devices on the same silicon chip exhibited the current recovery and the single‐molecule behavior, indicating reproducibility. The probability of molecule connection in a single device is ≈11% experimentally, and the probability of single molecule connection is ≈95% in the Section “Analysis of molecular connection” of the Supporting Information.

The transformation dynamics of crown ether ring at the single‐molecule level was monitored in a high vacuum chamber with precise temperature control. According to the charge transport theory, the conductance of the single‐molecule junction is closely related to its intrinsic structure.^[^ ^30^ ^]^

To monitor the structural changes of a single molecule, a high‐speed sampling rate (57600 Sa·s^−1^) was employed to capture current fluctuations at a constant source‐drain bias voltage. Cation (K^+^) capture experiments were conducted to confirm the connection of the CE ring in Section “Cation (K^+^) capture experiments” of the Supporting Information, which shows the conductivity change before and after the addition of cation (Figures S13−S16, Supporting Information).

A representative current trajectory of a single‐molecule junction at 150 K under the constant voltage of 0.05 V is shown in Figure 1C. The dwell times of the forward/reverse transformation process were further extracted by idealizing the long‐time *I*−*t* trajectories with quantify unknown biophysics software. The distribution of dwell times was fitted with a single‐exponential decay function, demonstrating the typical lifetime at the microsecond level. To further understand the origin of the current fluctuation, systematic control experiments were conducted and no apparent current fluctuations were observed before molecular connection (open circuit, Figure S17, Supporting Information) or on a graphene homojunction device (Figure S18, Supporting Information). This proves that the electric signal fluctuations only originate from the single‐molecule junction.

The theoretical calculations have revealed the existence of two structures for the CE ring that are dominated by the intramolecular through‐space interaction (TSI). The molecular dipole axial components are marked, which are in the same direction as the positive electric field (Figure 1D,E; Figure S19, Supporting Information). The configurations of the isolated molecules can be generated using the simulated annealing method, and the energy difference is listed in Table S1 (Supporting Information). It is necessary to explain that TSI denotes nonbonding, spatial, comprehensive interactions between different components of the molecule. There are different molecular orbital alignments and TSIs in these two structures, and no net charge transfer occurs during the switching. As shown in Figures 1F and S20 (Supporting Information), the molecular orbitals of CE‐I (Figure 1D) do not show the overlap between the crown ether ring and the molecular bridge, indicating the absence of TSI in this structure. In contrast, the presence of overlapped perturbed highest occupied molecular orbitals (*p*‐HOMOs) in the structure of CE‐II (Figure 1E,G) indicates a strong TSI between the naphthene unit and benzene rings located on the molecular bridge. Molecular orbitals (HOMO−1, HOMO, LUMO, LUMO+1) of CE‐I and CE‐II (Figure S20, Supporting Information) show apparent orbital overlap between the CE ring and the backbone of the molecular bridge. Orbital overlap contributes to intramolecular TSI between these two parts, thus stabilizing the whole structure. In addition, local transmission path calculations (Figure S21, Supporting Information) of CE‐I and CE‐II show that in the CE‐II structure, there are extra transmission paths from the main molecular bridge to the CE ring, which can be considered as extra interactions on two moieties of CE‐II. It is necessary to notice that the conductance of a single‐molecule device is judged by the transmission from one end of the electrode to the other, rather than from the main molecular bridge to the side group. The latter should be considered as extra interactions in the CE‐II structure. At the same time, we performed the non‐covalent interaction analysis (NCI) (Figures S22 and S23, Supporting Information) which have showed that the weak interaction between two moieties of CE‐II is larger than that of CE‐I, and the additional stabilizing effect is also greater. The strong TSI induces a pronounced distortion of the crown ether ring, increases the intramolecular tension, and further alters the molecular electronic characteristics. As a result, the conductance of CE‐II decreased, which can be verified by the transmission calculations (Figure 1B). Single‐molecule conductance can be affected by the alignment of molecular energy levels relative to the Fermi levels of the electrodes.^[^ ^1^ ^]^ The small energy difference between the transport channel and the Fermi level leads to a small potential barrier for the transport process, showing the higher conductance. Here, the charge transport is dominated by the *p*‐HOMOs of CE since they are proximate to the Fermi level of graphene electrodes (Figure S24, Supporting Information). In Figure S24 (Supporting Information), the x‐axis values (*E*–*E*_f_) of *p*‐HOMO peaks are smaller, indicating that *p*‐HOMOs are closer to the graphene Fermi level. Correspondingly, more proximate of the transmission peak to the electrode Fermi level indicates a higher conductance. Therefore, it can be concluded that the high‐conductance state is attributed to the CE‐I (Figure S25, Supporting Information), which does not involve TSI. Conversely, the low‐conductance state can be attributed to the CE‐II, which exhibits a strong TSI. The corresponding time intervals for each conductance state are extracted through idealized fitting in *I*−*t* trajectories. The average time intervals of CE‐I state are 360 ± 5 µs (Figure 1H) and CE‐II state are 240 ± 25 µs (Figure 1I).

### 2.2 Kinetic and Thermodynamic Analysis of Structural Transformation

**Figure 2:**
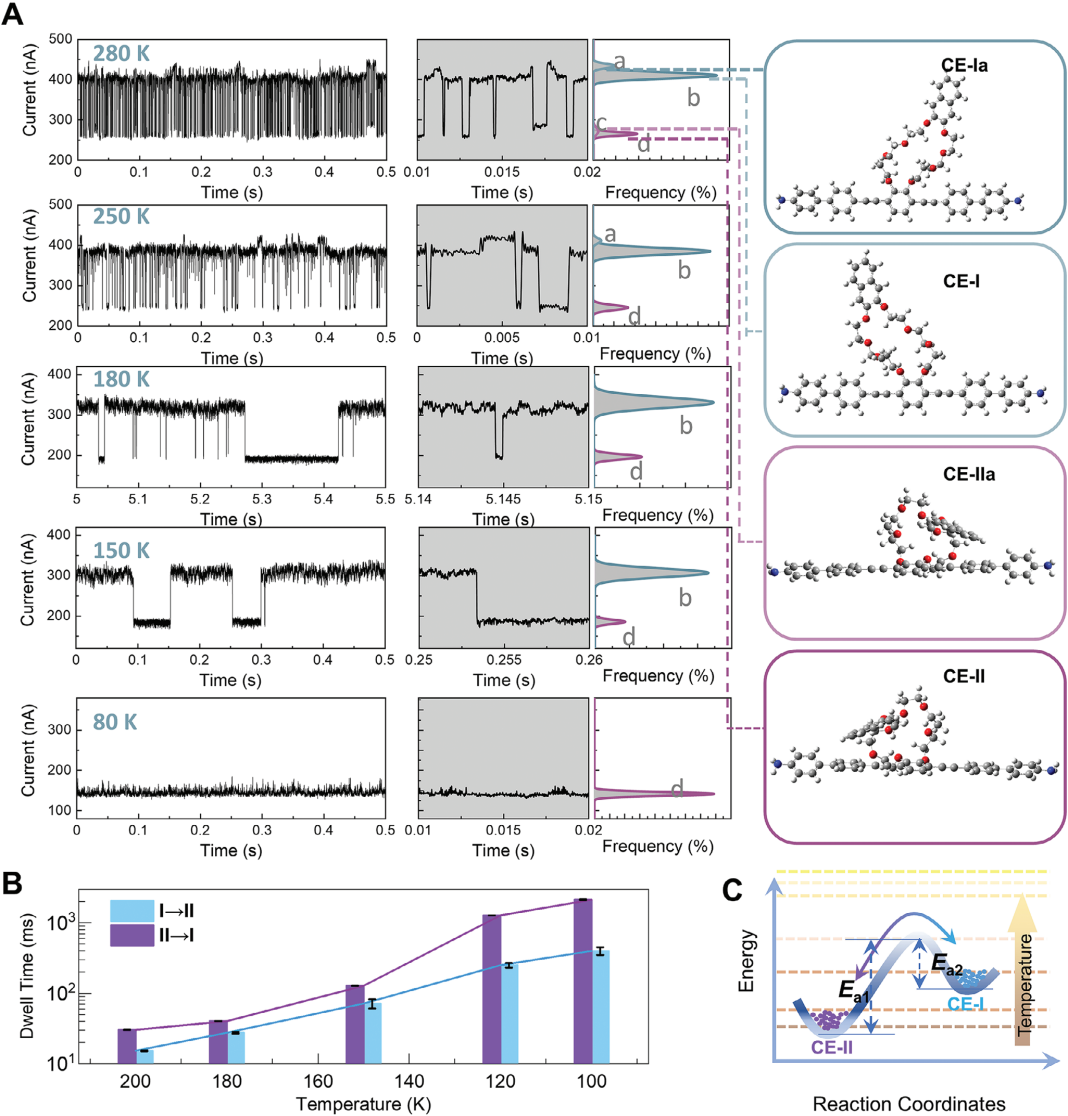
Temperature‐dependent structural transformations. A) Representative *I*−*t* trajectories at 0.05 V under different temperatures from 280 to 80 K and corresponding structures. B) Dwell times of the two tautomeric structures CE‐II and CE‐I from 200 to 100 K. The error scales are derived from the corresponding single exponential fittings of the dwell times in 3 different devices. C) The transformation energy diagram between CE‐I and CE‐II.

The kinetic and thermodynamic mechanisms of the structural transformation were revealed by monitoring the electrical signals under the temperature from 280 K to 80 K (**Figure** **2**A). The structural flipping between the CE‐I and CE‐II structures can be observed at temperatures at 280 K, which leads to the two new structures, CE‐Ia and CE‐IIa, as indicated by the Gaussian fitting of the conductance occupancy distribution. As the temperature decreased to 250 K, the conductance state of CE‐Ia disappeared; as the temperature decreased to 200 K, the conductance state of CE‐IIa disappeared. These two new states (CE‐Ia, CE‐IIa) at high temperatures are attributed mainly to the difference of conductance and energy. As mentioned above, the processes observed here are different spatial position orientations of the CE ring. For CE‐I and CE‐Ia, the conductance of these two states are relatively close, so it is speculated that the CE‐Ia is the “stand up” state of the CE ring in the opposite direction (Figure 2A). Since the molecular dipole of the “stand up” state in the opposite direction is anti‐parallel to the electric field direction, its stabilizing effect of the electric field is minimal. As a result, it exhibits a high transition barrier during testing, requiring high temperatures to observe the state. Similarly, for CE‐II, it can also be speculated that CE‐IIa is a similar “flat” state in the opposite direction (Figure 2A). It is necessary to notice that here the positive electric field direction is set from left to right. In addition, it should be noted that the symmetry of the molecular structure is broken due to the applied electric field, indicating that the two structures (CE‐I and CE‐Ia, CE‐II and CE‐IIa) are not completely mirror symmetric and show different transport characteristics. To further stabilize the molecular conformation, the temperature was further reduced below 100 K. It can be observed that the transformation between the CE‐II and CE‐I was inhibited and the twisted structure (CE‐II) was successfully stabilized (Figure 2A). With the decrease of temperature, the random tautomerization process is disrupted, and the “flat” structure of the crown ether can be stabilized to realize the efficient regulation.

The inherent lifetime and error bars of the corresponding process (Figure 2B) were extracted through single‐exponential fittings of the obtained dwell times from the long‐time monitoring (Figures S26 and S27, Supporting Information). For the forward process (CE‐II → CE‐I), the lifetime increased from ≈9 ms at 180 K to ≈2650 ms at 100 K, corresponding to a decrease in the forward reaction rate *k_f_* (*k* = 1/*τ*_avr_) from ≈111.1 to ≈0.37 s^−1^. Meanwhile, for the reverse process (CE‐I → CE‐II), the lifetime increased from ≈7 ms at 180 K to ≈350 ms at 100 K, indicating that the reverse reaction rate *k*_r_ decreased from ≈142.8 to ≈2.8 s^−1^. In addition, the free energy of activation can be calculated using the Eyring equation^22^ below:

(1) $$\Delta G^{‡}=-RT^{*}\ln\left(k\cdot h/k_{B}T\right)$$

where *R* is the gas constant, *h* is Planck's constant, and *k*_B_ is the Boltzmann constant. Plotting the reciprocals of temperatures against ln*k* (Figures S28 and S29, Supporting Information) showed a linear relationship, from which the activation energies of *E*_a1_ (∼1.94 kcal·mol^−1^, CE‐II → CE‐I) and *E*_a2_ (∼1.47 kcal·mol^−1^, CE‐I → CE‐II) are obtained. The energy diagram sketch of the transformation between CE‐II and CE‐I is shown in Figure 2C. Although the energy of the “flat” state (CE‐II) is less than that of the “stand up” state (CE‐I), at the micro level the “stand up” structure can be observed and stabilized by heat and external electric fields to overcome the structural tautomerization barrier. The increase in temperature can gradually overcome the flip barrier between the two states (Figure 2C), and effectively promote the transition between the two states. Through the interaction between the electric field and the molecular dipole, the energies of these two structures can be regulated under different electric fields, which in turn affects the proportion of these two structures.

### 2.3 Asymmetric Regulation of an External Electric Field

**Figure 3:**
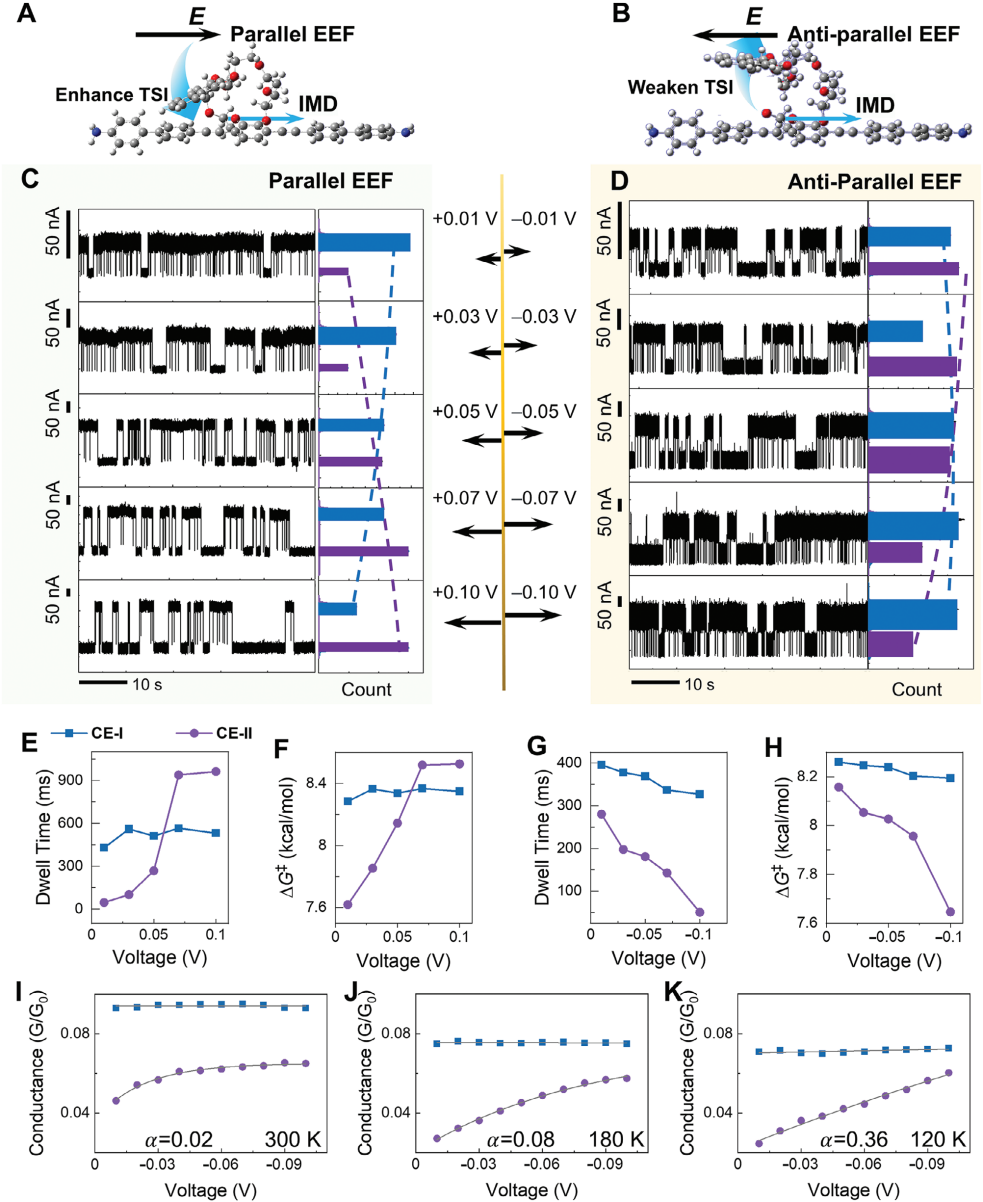
Asymmetric electric field regulation effects. A,B) Schematic of asymmetric electric‐gating effects resulting from parallel/anti‐parallel EEFs to IMD. C,D) *I*–*t* curves and corresponding histograms at different voltages of −0.01 −0.10 V and +0.01 +0.10 V at 150 K, respectively. The purple state is attributed to CE‐II and the blue state is attributed to CE‐I. E–H) The dwell times and activation‐free energies of the transformation between CE‐I and CE‐II under various external electric field strengths. I–K) Conductance changes of CE‐I and CE‐II under various external electric field strengths at different temperatures.

The strength of intramolecular TSI can be modulated through asymmetric electric regulation induced by the interactions between the external electric field (EEF) and the inherent molecular dipole (IMD). As shown in **Figure** **3**A,B, applying an anti‐parallel EEF was expected to decrease intramolecular TSI due to the increasing through‐space interaction distance, whereas applying a parallel EEF was expected to increase it. By observing the change of conductance states under the electric field in a certain direction, the direction of the molecular dipole can be analyzed. Considering the stability of different structures under the increasing electric field in a certain direction, the relative electric field direction to IMD of the molecule in the device can be obtained (parallel or anti‐parallel). The asymmetric electric‐regulating effect can be demonstrated by the dynamic analysis of voltage‐dependent experiments. As shown in Figure 3C,D, as the positive electric field strength increased, the occupancy of CE‐II (purple state) increased and the occupancy of CE‐I (blue state) decreased. Conversely, with an increase in negative electric field strength, the occupancy of CE‐II decreased and the occupancy of CE‐I increased. Here, the CE ring is regulated by the interaction between the electric field and the molecular dipole, the effect of current between source and drain on the CE ring is rather small because it is located in the side‐chain of the molecular bridge.

The energy difference between CE‐I and CE‐II in the parallel and anti‐parallel EEF was calculated in the Section “Calculated interaction energy under external electric fields” of the Supporting Information. In fact, the increase of the positive electric field or the decrease of the negative electric field will bring a stronger stabilization energy to CE‐II based on the dipole‐induced field effect and will produce different conformations under different electric field directions. Different directions of the electric field will exert different stabilizing effects on different molecular dipoles. When the direction of the EEF aligns with the IMD of the molecule, the state with a larger dipole moment becomes stabler as the electric field strength increases, leading to an increase in its proportion. Conversely, when the electric field is opposite to the intrinsic dipole moment, the state with a larger dipole moment possesses a weaker stabilization energy, causing the proportion to decrease as the electric field strength increases. After analyzing the proportion of a specific molecular state (e.g., CE‐II) under different electric fields, we can determine the relative orientation of the applied electric field to the intrinsic dipole moment of the molecule. Notably, the application of an external electric field is expected to increase the energy of the whole single‐molecule junction and generate symmetric effects under positive/negative EEFs, which is commonly observed in single‐molecule analysis.^[^ ^31^, ^32^ ^]^ However, the asymmetric effect^[^ ^33^, ^34^ ^]^ can be found in the device with intramolecular TSI by applying an external electric field.

Two aspects of the calculations were conducted to account for the effect of the electric field on molecular structure and TSI. On the one hand, from the perspective of energy, we approximately calculated the stabilization energy of CE ring with different configurations under an electric field in the Section “Calculated interaction energy under external electric field” of the Supporting Information. It can be found that as the CE ring changes from the CE‐I configuration to the CE‐II configuration, its axial dipole moment and the stabilizing effect of the electric field gradually increase. That is, an increase in parallel EEF would facilitate the transition from CE‐I to CE‐II, decrease the distance between two moieties and increase intramolecular TSI (Figure 3A). In addition, anti‐parallel EEF is expected to decrease intramolecular TSI due to the increasing through‐space interaction distance (Figure 3B). On the other hand, from the perspective of transmission, we calculated the molecular orbitals at different spacing distance between two moieties and found that the molecular transport channel *p*‐HOMO gradually approaches the Fermi level of graphene with the largen of the spacing (Table S3, Supporting Information), resulting in a gradual increase in the molecular conductance. These results are consistent with the experimental observation that the CE‐II conductance increases with the increase of the anti‐parallel electric field strength (or the decrease of the parallel electric field strength). This also suggests to some extent that the molecular CE‐II will gradually increase the distance (from the CE ring to the backbone) and the conductance with the increase of the anti‐parallel electric field strength.

Dwell times of the transformation processes between the two structures were extracted from the long‐term monitoring data. In the case of the CE‐II structure, a significantly prolonged dwell time can be observed, due to a more positive EEF strength (Figure 3E), and a rapid decrease in dwell time was associated with a much more negative EEF strength (Figure 3G). Conversely, for the CE‐I structure, the change in EEF‐dependent dwell time was far less conspicuous. The activation Gibbs energies, *∆G* ^‡^, extracted from the Eyring equation, showed that the energy barrier for the transformation from CE‐II to CE‐I increased with a rising positive EEF, but decreased with a larger negative EEF (Figure 3F,H). The energy barrier varied from ≈7.6 to ≈8.5 kcal·mol^−1^, within a 0.1 V voltage difference. In contrast, the change in the transformation barrier from CE‐I to CE‐II was very small (less than 0.2 kcal·mol^−1^, from ≈8.2 to ≈8.4 kcal·mol^−1^) within the same voltage range. This indicated that EEF mainly affects the CE‐II structure with a stabilizing effect under a positive EEF and a destabilizing effect under a negative EEF.

Theoretical calculations showed that the conductance of the single‐molecule junction with a fixed CE‐II structure decreased slightly with the increase of the EEF strength in both positive and negative directions without an asymmetric effect (Figures S30 and S31, Supporting Information). Experimentally, the conductance of CE‐I remained unchanged under negative EEFs, as shown in Figure 3I, which coincided with the calculation results. However, contrary to theoretical calculations, the conductance of CE‐II increased and gradually approached to that of CE‐I (Figure 3I–K; Figures S32 and S33, Supporting Information) at low temperatures. The disparity between theoretical computations and experimental findings primarily arises from the theoretical models. The CE‐II configuration was inherently constrained, whereas in experimental settings, an incremental elevation of the electric field induced a gradual structural transition of CE‐II toward CE‐I. Following an exponential relationship, *y*  =  *Ae* ^−*x*/α^, the conductance change of the CE‐II structure can be further fitted as a function of the bias voltage, where α represents the regulation factor. As the temperature increased, the regulation factor of CE‐II gradually decreased, suggesting that the EEF regulation became less effective. With the increase of temperature, the CE‐II structure was more profoundly influenced by the thermal fluctuations, enabling the structural transition among subtle configurations, thereby further diminishing the electric field's regulatory effect on the molecular structure.

### 2.4 The Mechanism for Quasi‐Continuous Regulation

**Figure 4:**
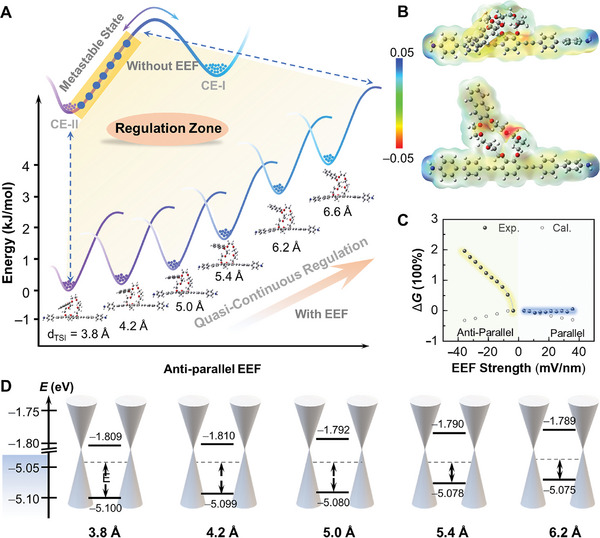
Mechanism analyses of quasi‐continuous regulation. A) Energy diagram for quasi‐continuous structural regulation of CE‐II under anti‐parallel EEFs. B) Electrostatic potential mappings of CE‐I and CE‐II. C) Theoretical and experimental conductance changes under parallel/anti‐parallel EEFs at 80 K. D) Molecular energy level characteristics for quasi‐continuous changing structures.

To understand the asymmetric electric field regulation mechanism, the quasi‐continuous characteristics of the CE‐II structure under different external electric fields are further analyzed. In the absence of an electric field, the single‐molecule junction can undergo free and random transitions between the CE‐I and CE‐II forms. When the parallel EEF is applied and increased, a higher energy of the CE‐II form is obtained, along with an increased distance between the naphthene moiety and the biphenyl moiety (Figure S34, Supporting Information), corresponding to a decreasing intramolecular TSI. In this circumstance, the EEF stabilizes the metastable structure of the molecule and generates a regulatable zone along the energy profile from CE‐II to CE‐I (**Figure** **4**A). The electrostatic potential mapping of the two states without an electric field is presented in Figure 4B, showing that the charges are mainly distributed in the flexible crown ether ring. The flexible structure and high charge density of the crown ether ring indicate that the structure can be easily regulated by an electric field. Thus, a series of metastable structures are quasi‐continuously stabilized by EEFs. Correspondingly, the HOMO/LUMO energy levels of the single‐molecule junction are quasi‐continuously modulated (Figure 4D; Figure S35, Supporting Information), ultimately leading to the movement of the molecular orbitals and reducing the energy level difference between the HOMO of the molecule and the Fermi level of graphene.

The conductance of the single‐molecule junction is influenced by the alignment between the frontier molecular energy level and the Fermi level of electrodes, which can be adjusted by the molecular dipole and intramolecular tension. The relative conductance difference between CE‐I and CE‐II becomes larger with higher parallel electric field strength, while it becomes smaller with higher anti‐parallel electric field strength. The Landauer–Büttiker transmission model^[^ ^35^ ^]^ demonstrates the feasibility of modulating the charge transport capability, and thereby the single‐molecule conductance, through adjusting the energy alignment between molecular orbitals and the Fermi level of electrodes (Table S3, Supporting Information). Furthermore, as the twisted molecular structure transits to the untwisted form, the molecular dipole gradually decreases (Figure S36, Supporting Information), and the internal molecular stress reduces. This, in combination with a more favorable energy level arrangement, enhances the conductivity of the single‐molecule junction. As shown in Figure 4C, experimental evidence demonstrates that the conductance of the CE‐II single‐molecule junction increases with the application of the negative electric field at 80 K. However, the conductance in the calculation is of the same structure under different electric field strengths, which is different from the experiment conditions. Theoretically, as the electric field strength increases, the conductive molecular orbitals in the transmission spectrum will move slightly away from the conductive window and the molecular conductance will decrease slightly.^[^ ^36^ ^]^ That is, the presence of a frontier orbital without structure changes results in a slight decrease in *G*, which is inappropriate in this experiment. In this work, considering the field effect induced by the molecular dipole, the electric field gradually induces the flexible crown ether molecule to change from CE‐II to CE‐I, resulting in a significant change in its molecular orbital. Therefore, the original conductance of CE‐II gradually increases to that of CE‐I. This phenomenon is a direct result of the efficient modulation of the crown ether structure.

## 3 Conclusion

In summary, this study demonstrates the efficient regulation of single‐molecule orbital properties through the interaction between the external electric field and the inherent molecular dipole. By controlling the direction and the strength of EEF, whether parallel or anti‐parallel to IMD, we can efficiently modulate the transformation barriers between CE‐I and CE‐II. In addition, the theoretical and experimental results prove that the flexible crown ether structure can be quasi‐continuously regulated under different EEF strengths. The charge distribution within the single‐molecule junction is redistributed under EEFs, which increases the energy of the single‐molecule junction, regulates the molecular orbitals, and stabilizes the meta‐stable structures. This research demonstrates the convenient and efficient use of EEF for modulating structural configurations and molecular orbitals of artificial macrocyclic molecule to contribute to the assembly and preset operation of molecular machines, which has potential applications in design of advanced materials, and the development of functional devices.

## Conflict of Interest

Authors declare no competing interests.

## Author Contributions

Z.L., W.H., S.Z., and C.X. contributed equally to this work. X.G., C.J. and L.Z. conceived the idea for the paper. Z.L. fabricated the devices and performed the device measurements. C.X. carried out the molecular synthesis. S.Z. and J.W. built and analyzed the theoretical model and performed the quantum transport calculation. X.G., C.J., Z.L., W.H., M.L., J.W. and S.H. analyzed the data and wrote the paper. All the authors discussed the results and commented on the manuscript.

## Supporting information



Supporting Information

## Data Availability

The data that support the findings of this study are available from the corresponding author upon reasonable request.
