# Supplementary material for: Quasi‐Continuous Efficient Regulation of Single‐Molecule Electronic Distribution
Source: Adv Sci (Weinh). 2025 Feb 19;12(14):2412260. doi: 10.1002/advs.202412260 (PMC11984856; doi:10.1002/advs.202412260)
Supplement: Supplementary file 1 — Supporting Information [file ADVS-12-2412260-s001.pdf]

ADVANCED SCIENCE
Open Access

Supporting Information

for *Adv. Sci.*, DOI 10.1002/advs.202412260

Quasi-Continuous Efficient Regulation of Single-Molecule Electronic Distribution

Zhizhou Li, Weilin Hu, Shuyao Zhou, Changqing Xu, Mingyao Li, Jinying Wang, Suhang He, Liang Zhang, Chuancheng Jia* and Xuefeng Guo**

Supplementary Information

Quasi-Continuous Efficient Regulation of Single-Molecule Electronic Distribution

Zhizhou Li[1], Weilin Hu[1], Shuyao Zhou[1], Changqing Xu[2], Mingyao Li[1], Jinying Wang[3], Suhang He[3], Liang Zhang[2], Chuancheng Jia[3]*, Xuefeng Guo[1,3]**

[1]Beijing National Laboratory for Molecular Sciences, National Biomedical Imaging Center, College of Chemistry and Molecular Engineering, Peking University, 292 Chengfu Road, Haidian District, Beijing 100871, P. R. China.

[2]School of Chemistry and Molecular Engineering, East China Normal University, Shanghai 200062, P. R. China.

[3]Center of Single-Molecule Sciences, Institute of Modern Optics, Frontiers Science Center for New Organic Matter, Tianjin Key Laboratory of Micro-scale Optical Information Science and Technology, College of Electronic Information and Optical Engineering, Nankai University, 38 Tongyan Road, Jinnan District, Tianjin 300350, P. R. China.

*Corresponding author. Email: guoxf@pku.edu.cn (X.G.); jiacc@nankai.edu.cn (C.J.); zhangliang@chem.ecnu.edu.cn (L.Z.)

Table of Contents:

Section 1: Material synthesis and characterization

Section 2: Device fabrication and characterization

Section 3: Theoretical calculation and analysis

Schemes S1–S3

Figures S1–S36

Tables S1–S3

References

Section 1: Material synthesis and characterization

1.1 General Experimental Section

Unless otherwise stated, all starting materials and anhydrous solvents were obtained from commercial suppliers and were used without further purification. All moisture- or air-sensitive reactions were performed using oven-dried glassware under an inert atmosphere of dry argon. Air- or moisture-sensitive liquids and solutions were transferred via syringe. ^{1}H NMR and ^{13}C NMR were measured on Bruker 400 MHz Spectrometer. Chemical shifts were reported in parts per million (ppm) downfield from high to low frequency using the residual solvent peak as the internal reference ($CDCl_3$ = 7.26 ppm; DMSO-d_6 = 2.50 ppm). Correlation spectroscopy (COSY), heteronuclear single quantum coherence (HSQC) and heteronuclear multiple bond correlation (HMBC) experiments were used to aid structural determination and spectral assignment. Flash column chromatography was carried out using Silica 60 Å (particle size 40-63 μm) as the stationary phase. Visualized using both short and long wave ultraviolet light. Mass spectrometry was carried out by the Mass Spectrometry Service Centre at School of Chemistry and Molecular Engineering, East China Normal University.

1.2 Abbreviations

DMSO	Dimethylsulfoxide
DMF	*N*,*N*-Dimethylformamide
TEA	Triethylamine
MeCN	Acetonitrile
TsCl	*p*-Toluenesulfonyl chloride
r.t.	room temperature
THF	Tetrahydrofuran
MeOH	Methanol
DMAP	4-Dimethylaminopyridine
DCM	Dichloromethane
aq.	Aqueous

1.3 Synthetic Overview

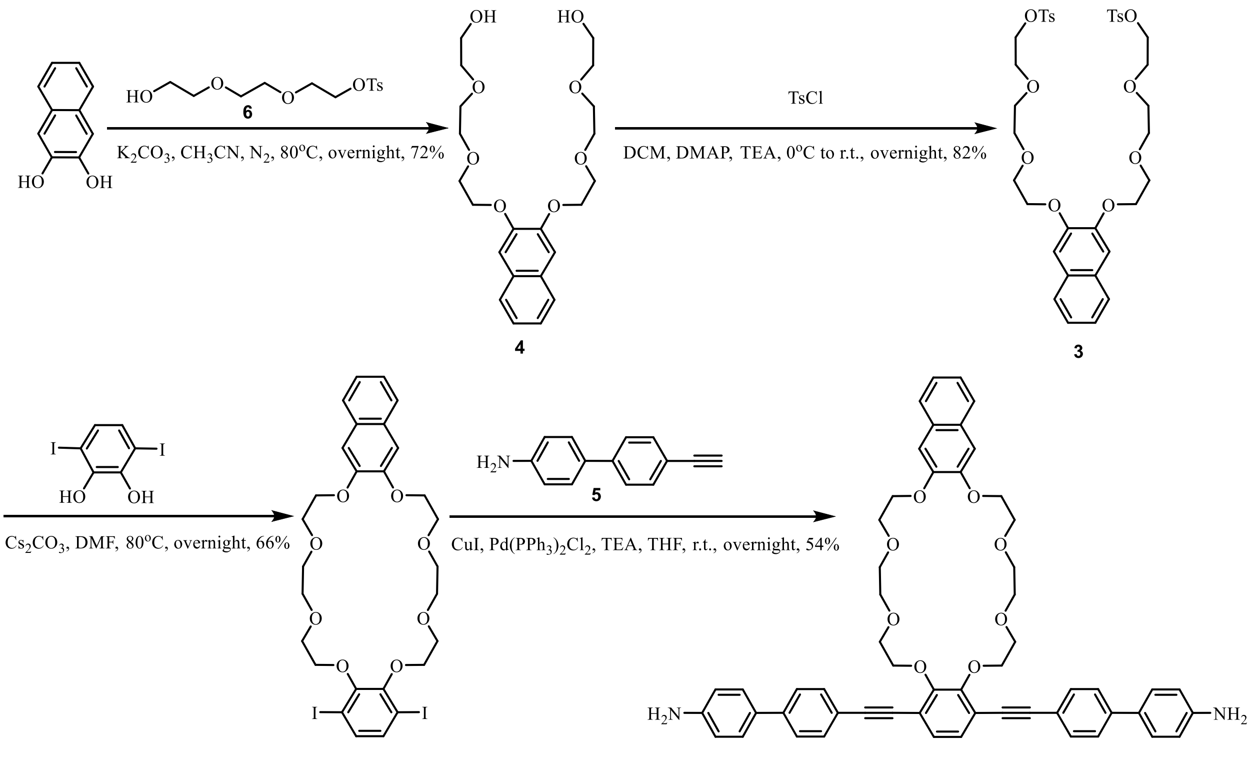

Scheme S1: Synthesis of macrocycle 1. Compounds **5**, **6** were prepared as previously reported.[1]

1.4 Synthetic Procedures and Characterization Details

Synthesis of compound 2

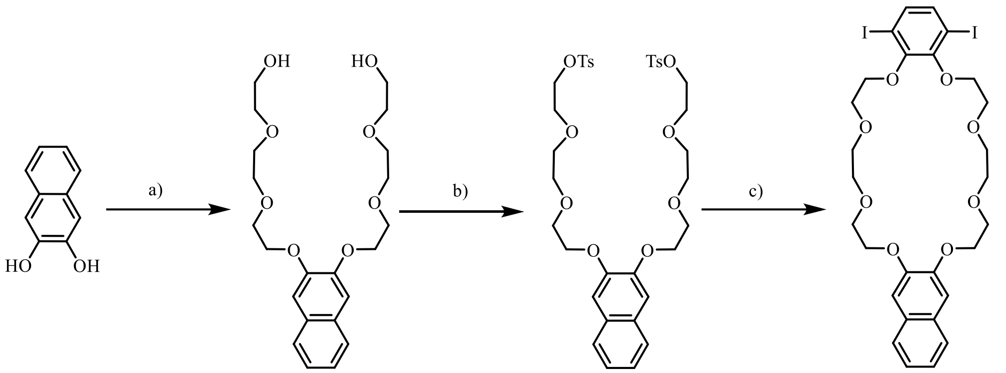

4 **3** **2**

Scheme S2: Synthesis of 2. Compound 3,6-Diiodo-1,2-benzenediol was prepared as described previously.[1] Reagents and conditions: a) 2,3-Dihydroxynaphthalene, **6**, K_2CO_3, CH_3CN, 80°C, overnight, 72%; b) TEA, DCM, DMAP, 0°C to r.t., overnight, 82%; c) 3,6-Diiodo-1,2-benzenediol, Cs_2CO_3, DMF, 80°C, overnight, 66%.

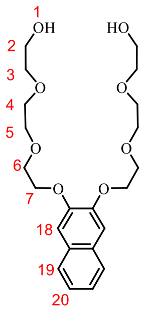

2,3-Dihydroxynaphthalene (478 mg, 3 mmol), K_2CO_3 (1.2 g, 8.96 mmol) and **6** (2 g, 6.6 mmol) in dry CH_3CN, was stirred overnight under N_2 at 80 °C. The reaction mixture was filtered and then concentrated under reduced pressure. The crude product was purified by column chromatography over silica gel with DCM/MeOH (100:5), **4** is obtained in ~72% yield as light-yellow oil. ^1H NMR (400 MHz, $CDCl_3$, 298 K) δ 7.65 (dd, J = 6.1, 3.3 Hz, $2H_{19}$), 7.32 (dd, J = 6.1, 3.2 Hz, $2H_{20}$), 7.13 (s, $2H_{18}$), 4.31 – 4.25 (m, $4H_7$), 3.99 – 3.93 (m, $4H_6$), 3.80 – 3.75 (m, $4H_3$), 3.74 – 3.67 (m, $8H_{4,5}$), 3.63 – 3.57 (m, $4H_2$). ^{13}C NMR (101 MHz, $CDCl_3$, 298 K) δ 148.66, 129.29, 126.34, 124.34, 108.13, 72.82, 70.89, 70.27, 69.44, 68.14, 61.67. MS (ESI^+): Calcd. For $C_{22}H_{33}O_8^+$: 425.2170, found 425.3604 $[M+H]^+$.

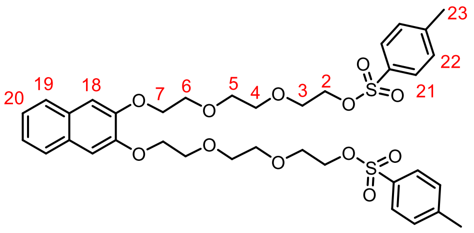

The solution of **4** (0.9 g, 2.1 mmol), TEA (1.5 mL, 10.6 mmol), TsCl (1.6 g, 8.5 mmol) and DMAP (18 mg, 0.2 mmol) in DCM was stirred at 0°C for 30min, then warmed up to room-temperature and stirred overnight. The reaction mixture was filtered and then concentrated under reduced pressure. The crude product was purified by column chromatography over silica gel with DCM/MeOH (100:5), **3** is obtained in ~82% yield as light-yellow solid. ^{1}H NMR (400 MHz, $CDCl_3$, 298 K) δ 7.77 (d, J = 8.4 Hz, 4H_{21}), 7.66 (dd, J = 6.1, 3.3 Hz, 2H_{20}), 7.36 – 7.27 (m, 6$H_{19,22}$), 7.14 (s, 2H_{18}), 4.27 – 4.22 (m, 4H_7), 4.17 – 4.12 (m, 4H_2), 3.91 (dd, J = 5.6, 4.3 Hz, 4H_6), 3.73 – 3.67 (m, 8$H_{4,5}$), 3.64 – 3.59 (m, 4H_3), 2.40 (s, 6H_{23}). ^{13}C NMR (101 MHz, $CDCl_3$, 298 K) δ 148.98, 144.80, 132.99, 129.83, 129.36, 127.96, 126.34, 124.28, 108.54, 70.88, 70.85, 69.63, 69.30, 68.73, 68.40, 21.61. MS (ESI$^+$): Calcd. For $C_{36}H_{45}O_{12}S_2^+$: 733.2347, found 733.2324 $[M+H]^+$.

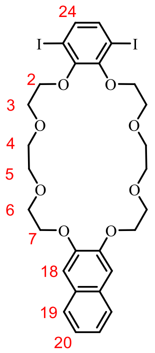

The solution of 3,6-diiodo-1,2-benzenediol (565 mg, 1.6 mmol), Cs_2CO_3 (2.54 g, 7.8 mmol) in DMF was stirred at 80°C for 30 min under N_2, then a solution of **3** (1.15 g, 1.56 mmol) in DMF was added into the mixture slowly then stirred overnight. The reaction mixture was filtered and then concentrated under reduced pressure. The crude product was purified by column chromatography over silica gel with DCM/MeOH (100:5), **2** is obtained in~ 66% yield as light-yellow solid. ^{1}H NMR (400 MHz, $CDCl_3$, 298 K) δ 7.65 (dd, J = 6.1, 3.3 Hz, 2H_{20}), 7.32 (dt, J = 6.2, 3.4 Hz, 2H_{19}), 7.21 (s, 2H_{24}), 7.12 (s, 2H_{18}), 4.30 – 4.25 (m, 4H_7), 4.22 – 4.16 (m, 4H_2), 4.04 – 3.99 (m, 4H_6), 3.97 – 3.92 (m, 4H_3), 3.89 (dd, J = 5.9, 3.5 Hz, 4H_5), 3.81 (dd, J = 5.8, 3.5 Hz, 4H_4). ^{13}C NMR (101 MHz, $CDCl_3$, 298 K) δ 152.45, 149.13, 135.47, 129.36, 126.32, 124.21,

108.34, 93.15, 72.68, 71.24, 70.96, 70.51, 69.79, 69.14. MS (ESI$^+$): Calcd. for $C_{28}H_{33}I_2O_8^+$: 751.0260, found 751.0646 [M+H]$^+$.

Synthesis of compound macrocycle 1

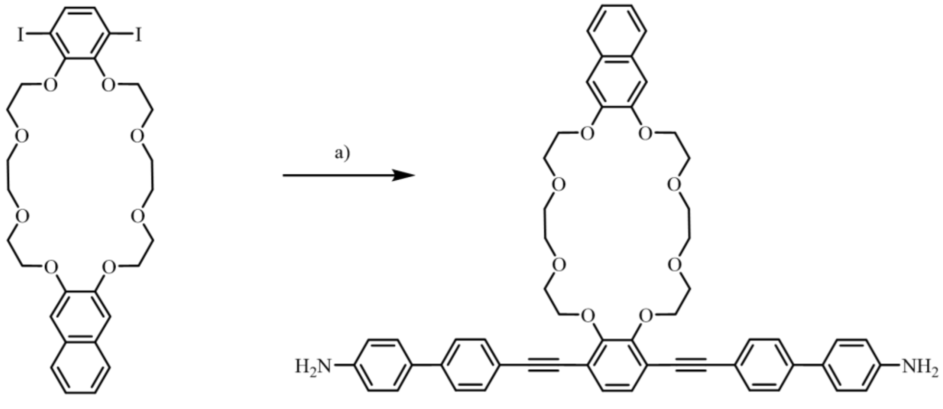

1

Scheme S3: Synthesis of macrocycle 1. Reagents and conditions: a) **5**, CuI, $Pd(PPh_3)_2Cl_2$, TEA, THF, r.t., overnight, 54%.

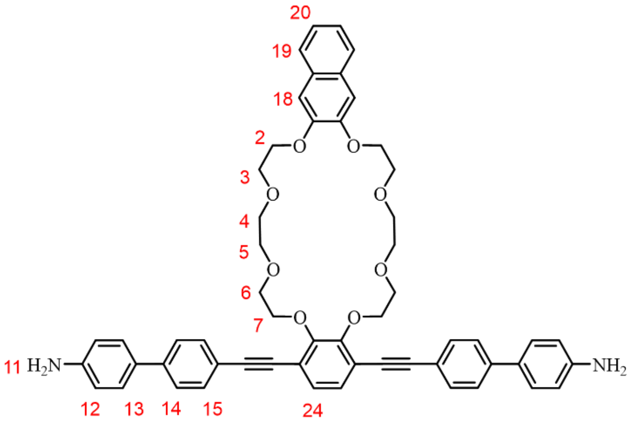

CuI (16.5 mg, 0.086 mmol) and $PdCl_2(PPh_3)_2$ (60.6 mg, 0.086 mmol) are added into a solution of **2** (500 mg, 0.67 mmol) in THF at room-temperature under N_2, then 1.9 mL TEA was injected into the solution and stirred for 10 min. The solution of **5** (386.3 mg, 2.0 mmol) in THF was then injected into the solution slowly. Stirred overnight at room-temperature. Dealt with NH_4Cl (aq.) and extracted with H_2O/DCM, keep the organic phase and then concentrated under reduced pressure. The crude product was purified by column chromatography over silica gel with DCM/MeOH (100:5) and get macrocycle **1** in ~54% yield as yellow solid. ^{1}H NMR (400 MHz, DMSO-d_6, 298 K) δ 7.71 (dd, J = 6.2, 3.4 Hz, $2H_{20}$), 7.61 (d, J = 8.1 Hz, $4H_{15}$), 7.53 (d, J = 8.0 Hz,

$4H_{14}$), 7.42 (d, J = 8.3 Hz, $4H_{13}$), 7.30 (d, J = 4.6 Hz, $4H_{18,19}$), 7.25 (s, $2H_{24}$), 6.66 (d, J = 8.2 Hz, $4H_{12}$), 5.36 (s, $4H_{11}$), 4.36 – 4.26 (m, $4H_7$), 4.24 – 4.15 (m, $4H_2$), 3.86 (q, J = 5.2 Hz, $8H_{3,6}$), 3.77 – 3.66 (m, $8H_{4,5}$). ^{13}C NMR (101 MHz, DMSO-d_6, 298 K) δ 153.47, 149.48, 149.10, 141.56, 132.24, 129.39, 128.03, 127.74, 126.67, 126.46, 125.81, 124.38, 119.54, 118.89, 114.69, 108.24, 95.93, 86.19, 73.55, 70.80, 70.58, 70.49, 69.44, 68.98. MS (ESI$^+$): Calcd. for $C_{56}H_{52}N_2O_8Na^+$: 903.3616, found 903.3880 [M+Na]$^+$.

1.5 ^{1}H and ^{13}C NMR Spectra

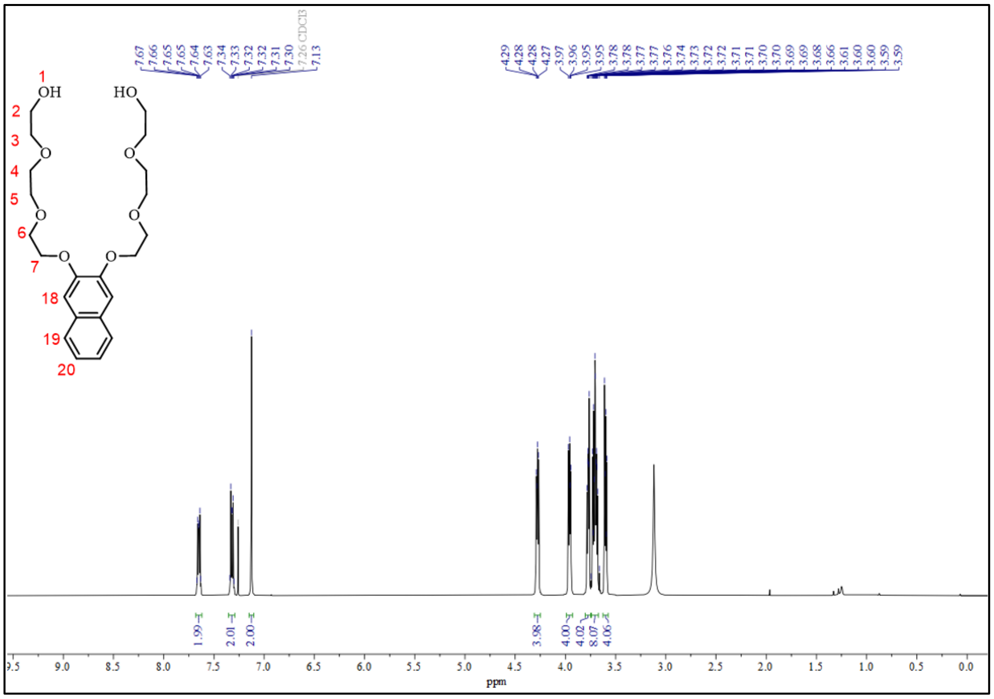

Figure S1: ^{1}H NMR (400M, $CDCl_3$, 298 K) spectrum of compound 4.

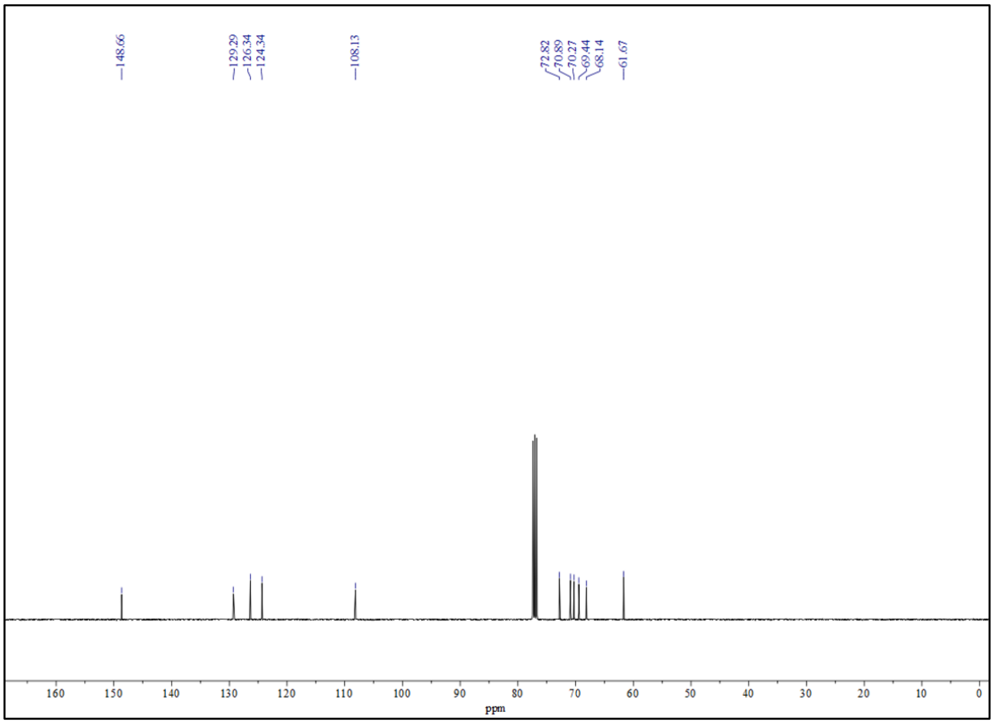

Figure S2: ^{13}C NMR (101M, $CDCl_3$, 298 K) spectrum of compound 4.

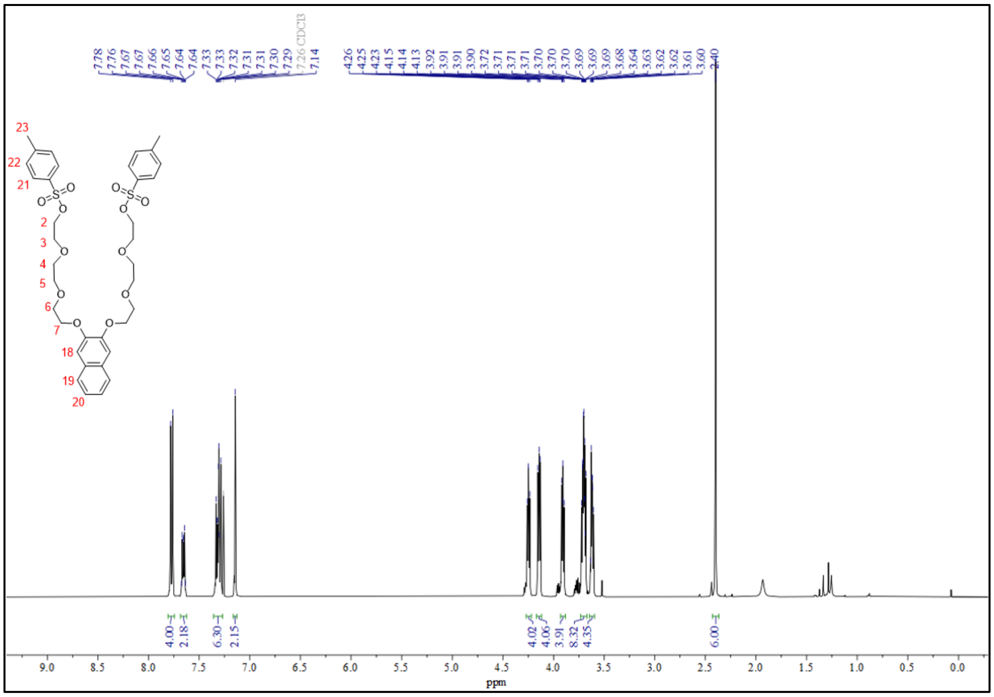

Figure S3: ^{1}H NMR (400M, $CDCl_3$, 298 K) spectrum of compound 3.

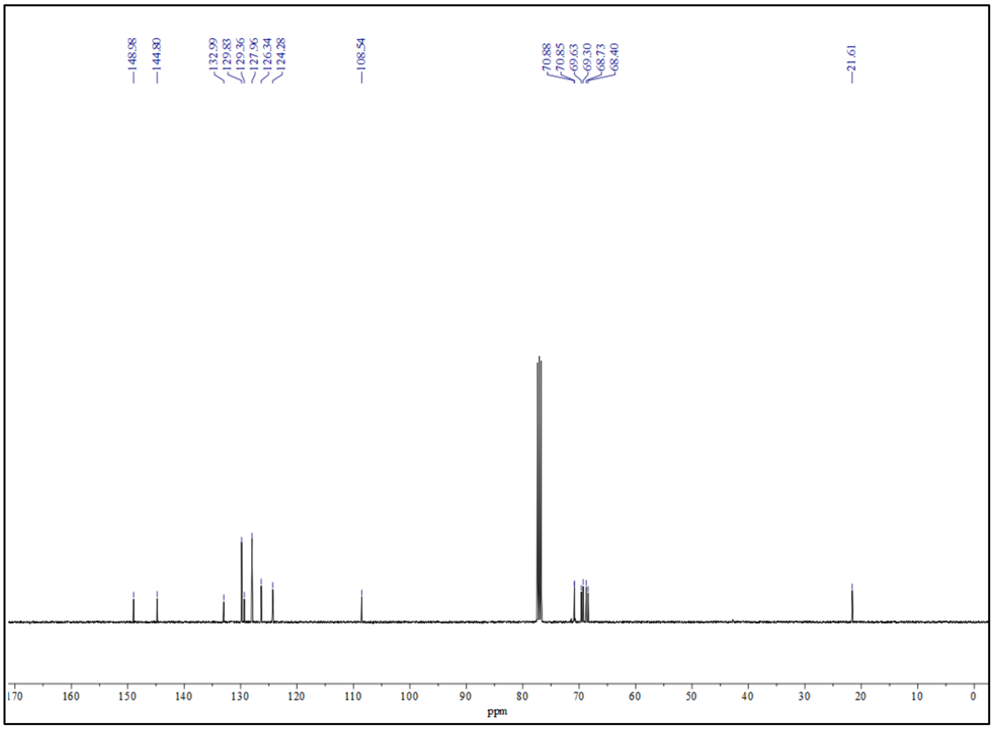

Figure S4: ^{13}C NMR (101M, $CDCl_3$, 298 K) spectrum of compound 3.

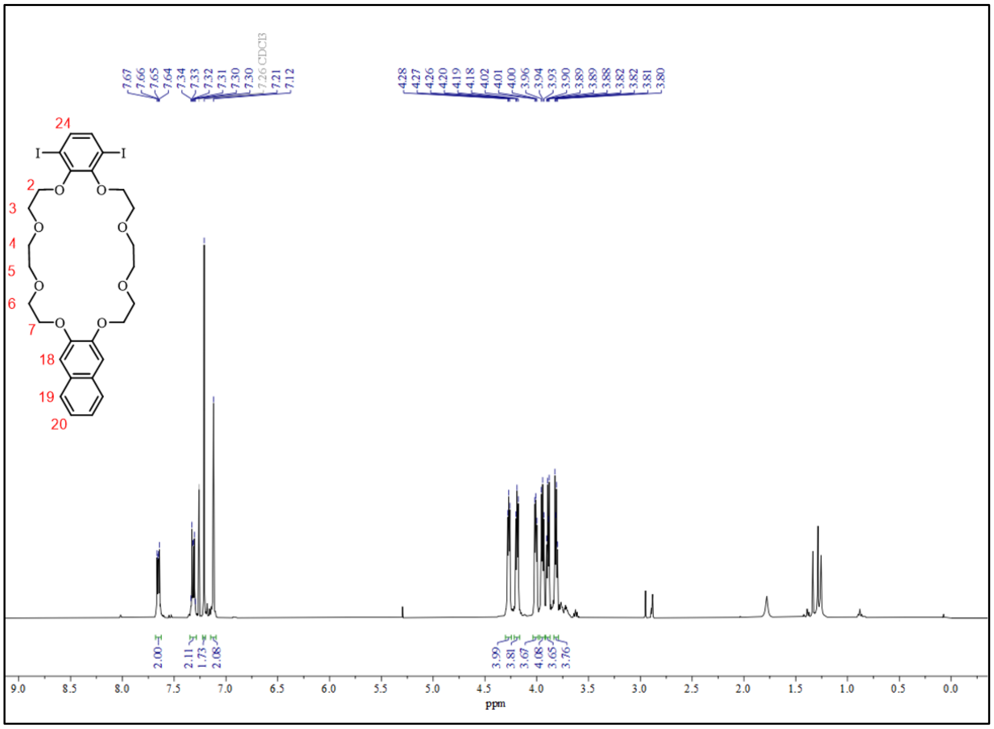

Figure S5: ^{1}H NMR (400M, $CDCl_3$, 298 K) spectrum of compound 2.

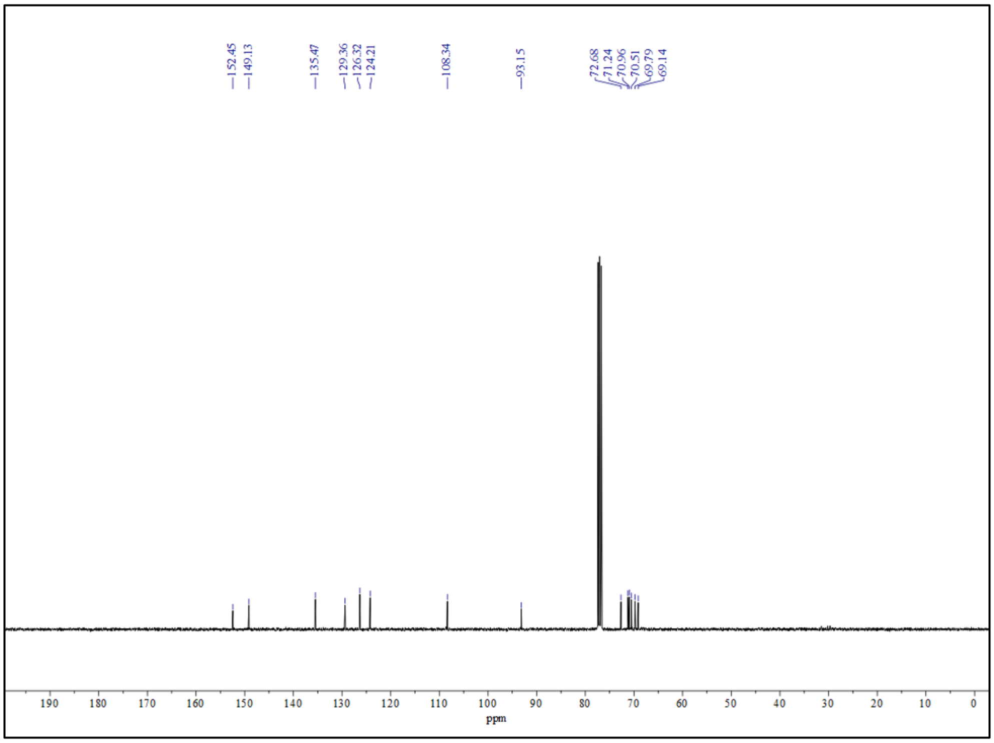

Figure S6: ^{13}C NMR (101M, $CDCl_3$, 298 K) spectrum of compound 2.

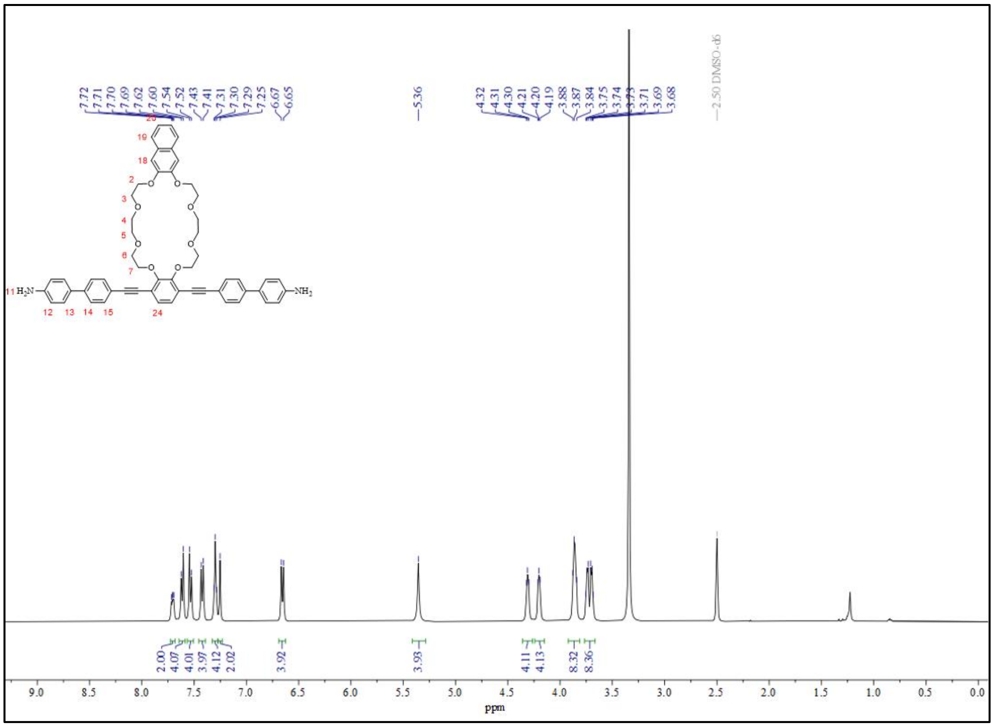

Figure S7: ^{1}H NMR (400M, DMSO-d_6, 298 K) spectrum of compound 1.

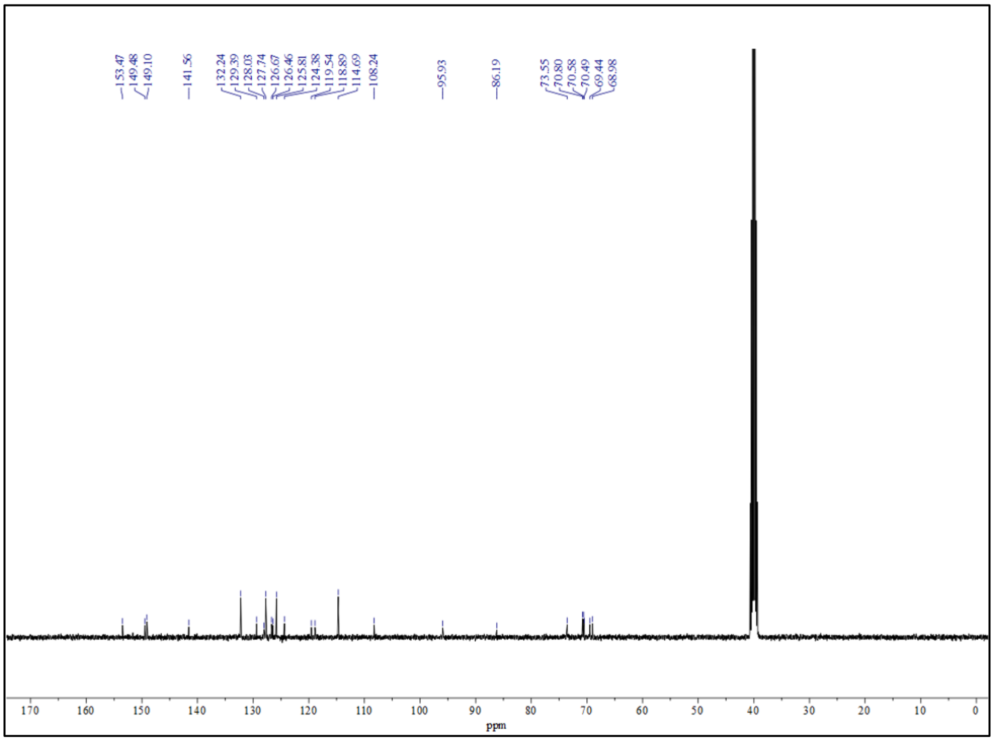

Figure S8: ^{13}C NMR (101M, DMSO-d_6, 298 K) spectrum of compound 1.

Section 2: Device fabrication and characterization

Device fabrication and molecular connection.

The point-contact graphene electrodes for single-molecule devices were prepared using the dash-line lithography (DLL) method, as previously described.[2] To establish molecular connections, the graphene devices, crown ether compounds, and 1-ethyl-3-(3-dimethylaminopropyl) carbodiimide hydrochloride (EDCI) were introduced into a reaction vessel under an argon (Ar) protective atmosphere to exclude moisture and oxygen. Subsequently, 10 mL of anhydrous pyridine was injected into the vessel to create a molecular solution with a concentration of approximately 10^{-4} M. Following a two-day incubation period in dark to allow for molecular connections, the devices were removed from the solution. Then, the devices were cleaned with acetone and ultrapure water to eliminate surface residues. Finally, the devices were dried using N_2 gas.

Device characterization

By using a Keysight B1500A semiconductor characterization system and a Karl Suss (PM5) manual probe station, the devices were characterized at room temperature in the ambient atmosphere. The temperature-dependent electrical characterization of the devices was performed on Lakeshore TTPX Cryogenic Probe Stage with a Model 336 Controller with the liquid nitrogen cooling system. The *I*–*t* measurements were carried out from 80 K to 300 K in a vacuum environment. The applied constant voltage was established, utilizing the auxiliary output of the UHFLI lock-in amplifier, while the current signal was amplified, using a DL1211 amplifier. To facilitate the data recording process, a high-speed acquisition card (NIDAQ) was employed, enabling data acquisition at a rate of 57,600 samples per second.

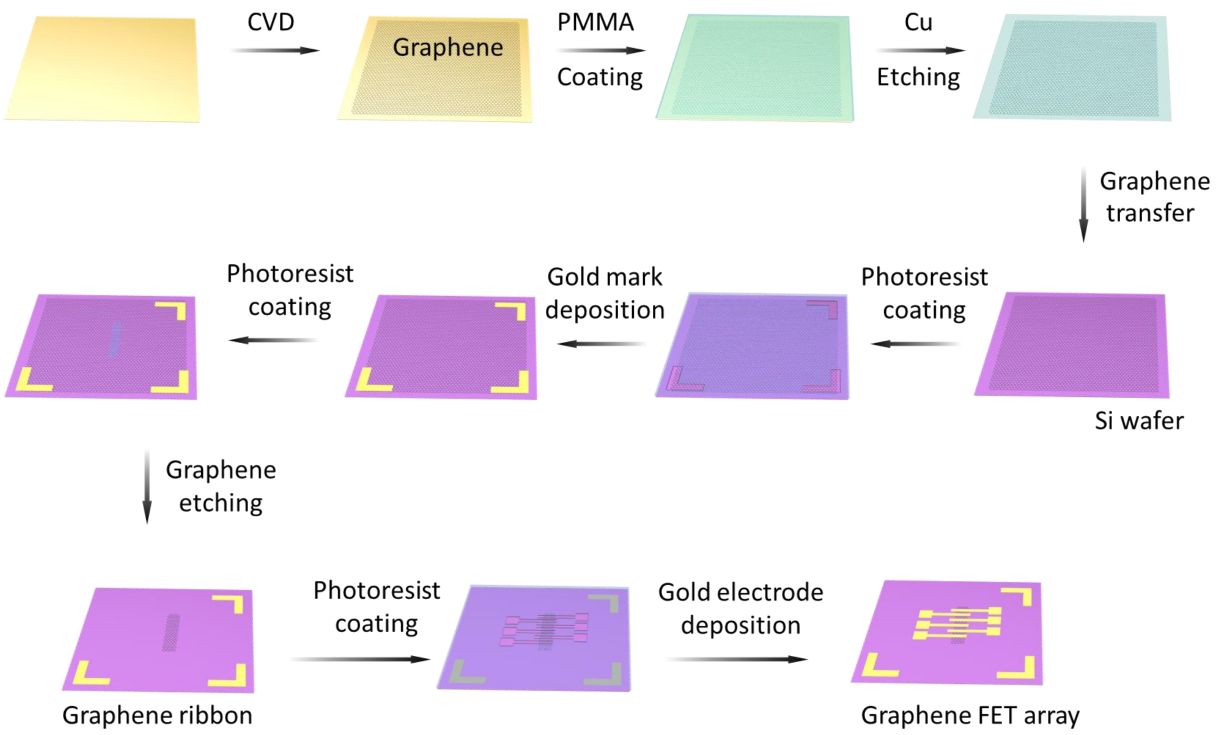

Figure S9: Fabrication of graphene field-effect transistors (FETs) with Cr (8 nm) /Au (60 nm) electrodes.

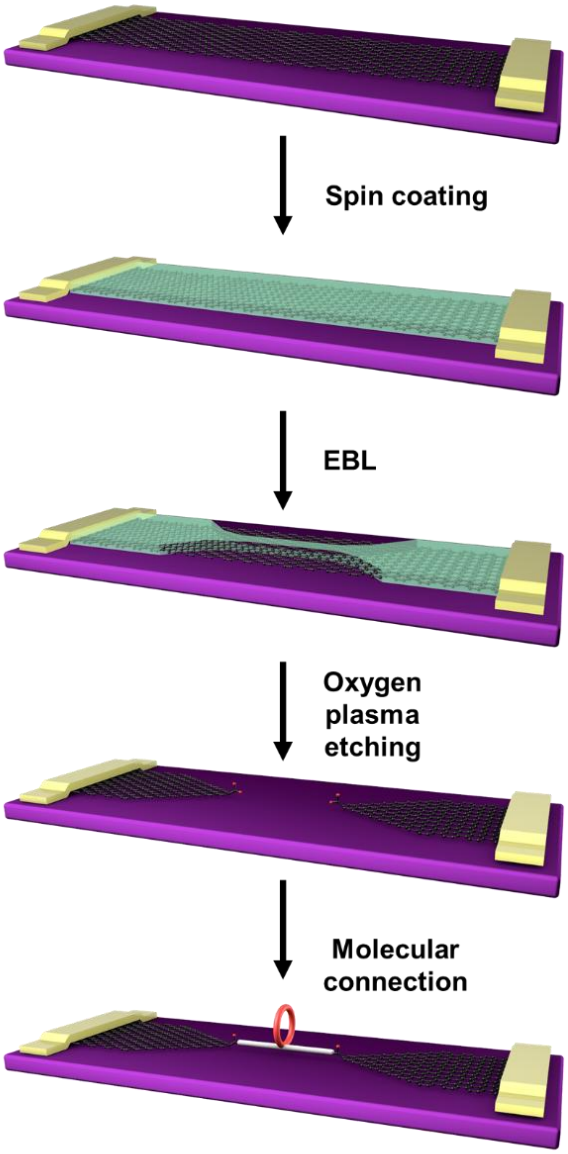

Figure S10: Schematic of the fabrication procedure of single-molecule devices. A single crown ether molecule is assembled into the pair of graphene electrodes.

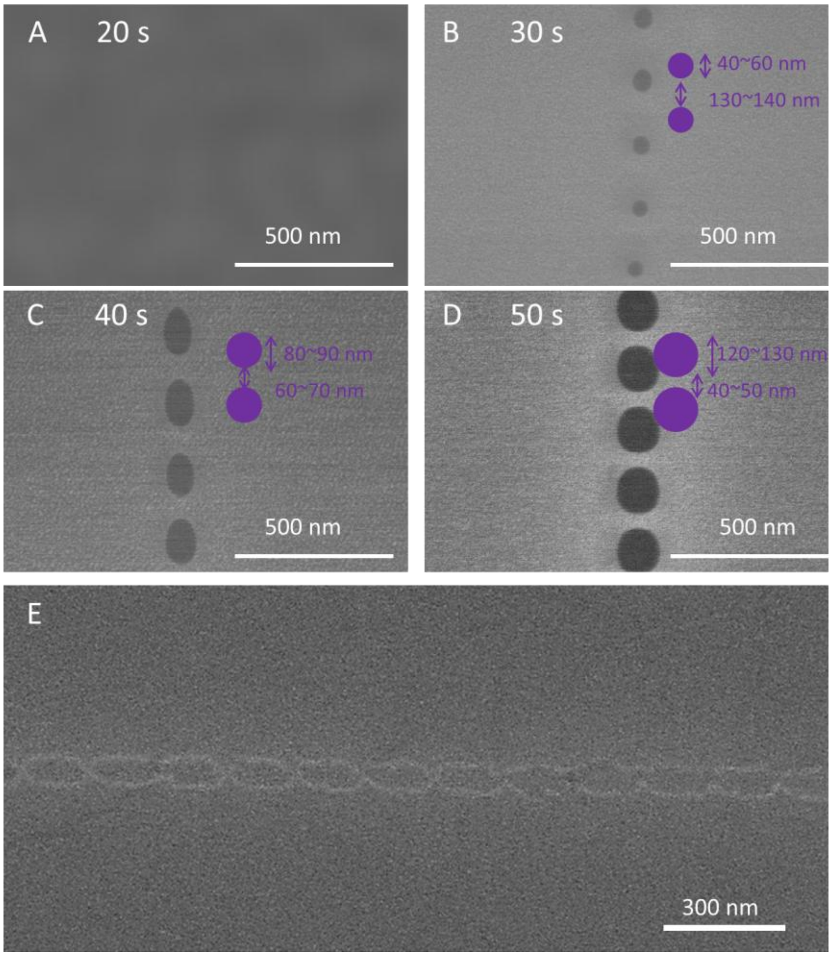

Figure S11: Scan electron microscope (SEM) images of the etching process. (**A–D**) The PMMA film after EBL and etching for different times. (**E**) The graphene nanoelectrode before molecular connection.

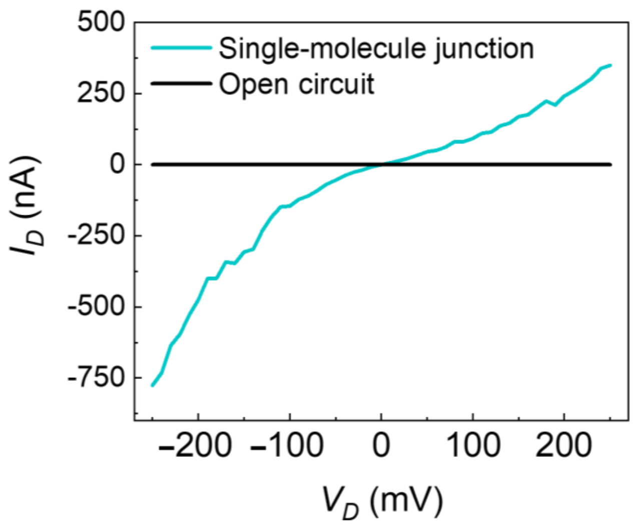

Figure S12: *I*−*V* curves for the device before and after molecular connection. The successful preparation of the single-molecule junctions is determined by comparing the current-voltage (*I*−*V*) relation curve after oxygen plasma etching (open circuit) with the curve after connecting the molecule (recovered to some extent).

Analysis of molecular connection

m = 210 pairs of graphene electrodes were prepared between each pair of gold electrodes *via* electron beam lithography (EBL). The probability of molecules being reconnected in a single device is ~11% experimentally, so the probability of a successful integration between a pair of graphene electrodes is ρ = 11%/210 = 0.052%.

The binomial distribution indicates the probability that *n* molecules are integrated to a pair of gold electrodes as:

$$G_n = \frac{m!}{n!\,(m-n)!}\rho^n(1-\rho)^{m-n}$$

Then, the probability of molecules being integrated to a pair of gold electrodes is:

$$\gamma_c = 1 - G_0 = 1 - \frac{m!}{0!\,(m-0)!}\rho^0(1-\rho)^m = 1-(1-\rho)^m$$

Thus, the probability of connecting only one molecule is: G_1/γ_c = ~95%.

This result indicates that in most cases, there is only one molecular integration between a pair of gold electrodes.

Cation (K^+) capture experiments

There was no switching of "stand up" and "flat" states during the test after K^+ addition. It is speculated that Van der Waals interaction in the original structure contributes to the switching process. When K^+ are captured in the ring, extra electrostatic effect might destroy the balance of these two states. Also, high temperature and bias-voltage prevents the observation of subtle structure changes.

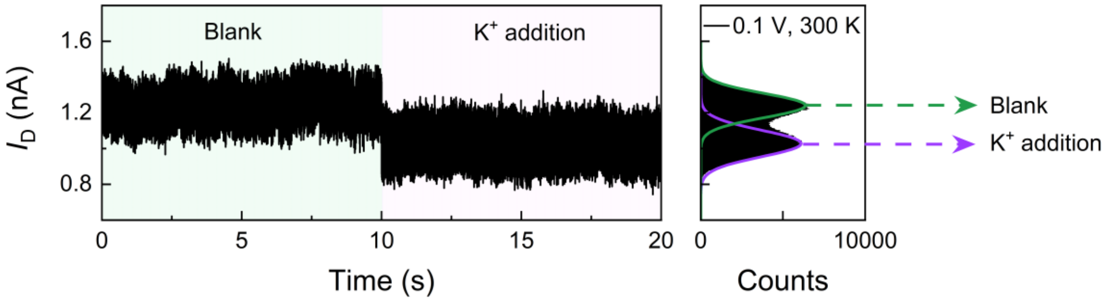

Figure S13: Cation (K^+) capture current–time experiment at 300 K with the bias of 0.1 V. The crown ether-based single-molecule junction was immersed in acetonitrile solution of potassium hexafluorophosphate (KPF_6). Left panel: 0–10 s of the current–time experiment represents the dry device (Blank), while the 10–20 s data represents the device with K^+ solution (K^+ addition). Right panel: histograms of the current in the dry device (Blank) and the device with K^+ solution (K^+ addition), which can be distinguished easily.

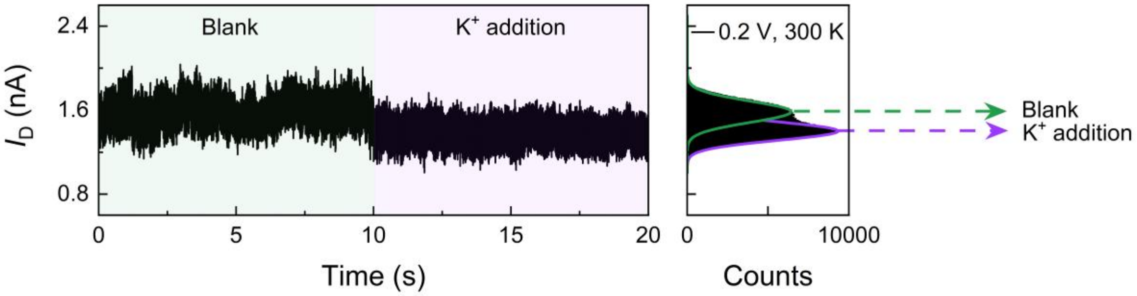

Figure S14: Cation (K^+) capture current–time experiment at 300 K with the bias of 0.2 V. The crown ether-based single-molecule junction was immersed in acetonitrile solution of potassium hexafluorophosphate (KPF_6). Left panel: 0–10 s of the current–time experiment represents the dry device (Blank), while the 10–20 s data represents the device with K^+ solution

(K^+ addition). Right panel: histograms of the current in the dry device (Blank) and the device with K^+ solution (K^+ addition), which can be distinguished.

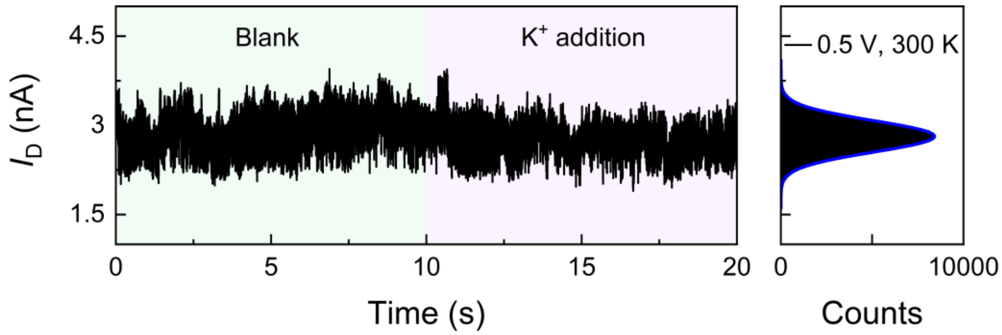

Figure S15: Cation (K^+) capture current–time experiment at 300 K with the bias of 0.5 V. The crown ether-based single-molecule junction was immersed in acetonitrile solution of potassium hexafluorophosphate (KPF_6). Left panel: 0–10 s of the current–time experiment represents the dry device (Blank), while the 10–20 s data represents the device with K^+ solution (K^+ addition). Right panel: histograms of the current in the dry device (Blank) and the device with K^+ solution (K^+ addition), which cannot be distinguished.

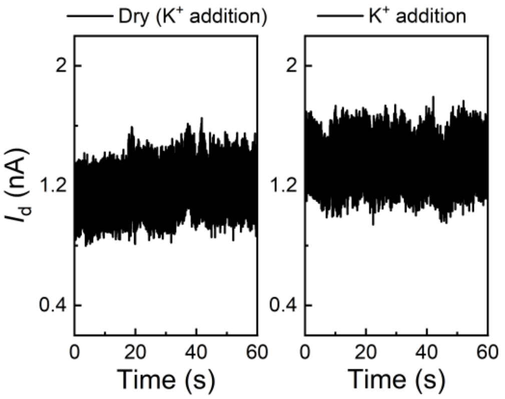

Figure S16: Cation (K^+) capture current–time experiment at 300 K with the bias of 0.2 V. The crown ether-based single-molecule junction was immersed in acetonitrile solution of potassium hexafluorophosphate (KPF_6). To exclude the effect of the addition of solution on the conductance change, the device with acetonitrile solution of KPF_6 was blow-dried. The conductance under solution was slightly higher than that in the dry state, which can exclude the influence of solution addition on the ion trapping experiments. Left panel: current–time data of the

blow-dried device with dropping acetonitrile solution of KPF_6. Right panel: current–time data of the device with dropping acetonitrile solution of KPF_6.

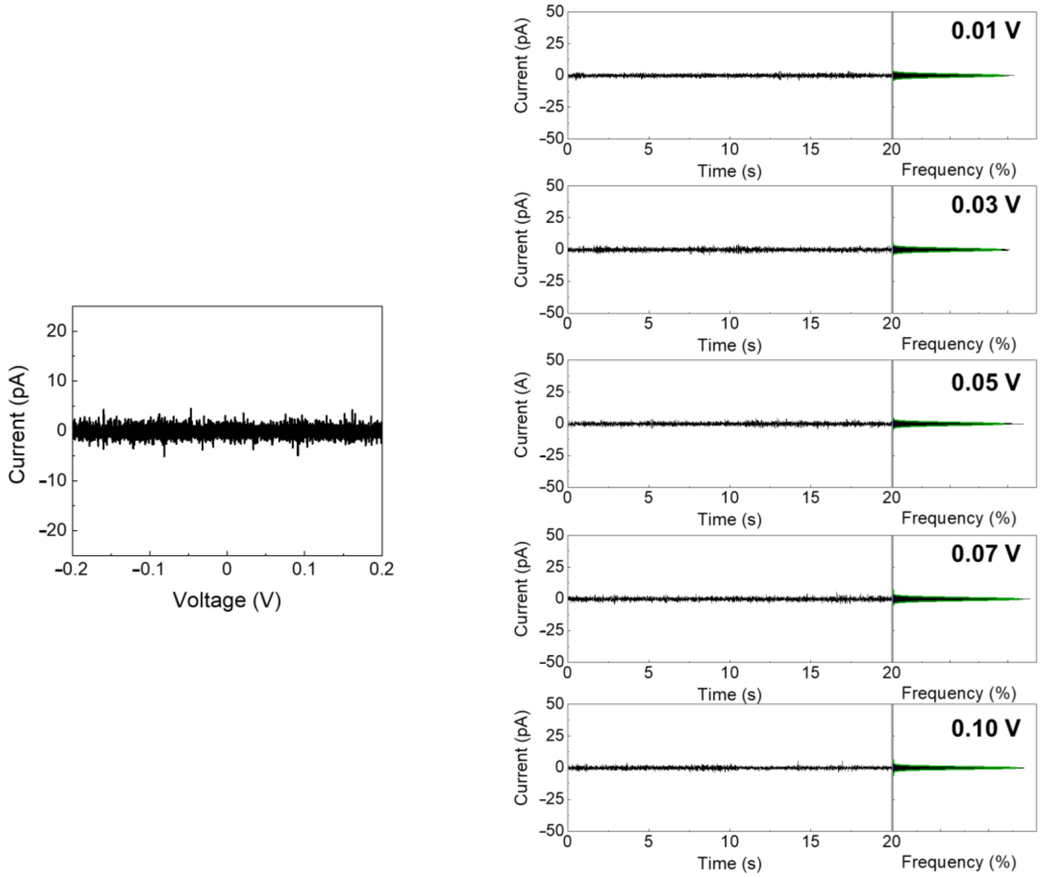

Figure S17: The current monitoring of the device before molecular connection at different voltages. Left panel: *I*–*V* curve of the device before molecular connection; Right panel: representative *I*–*t* trajectories and the corresponding histograms of the junction at 300 K and different voltages of 0.01 V, 0.03 V, 0.05 V, 0.07 V and 0.10 V.

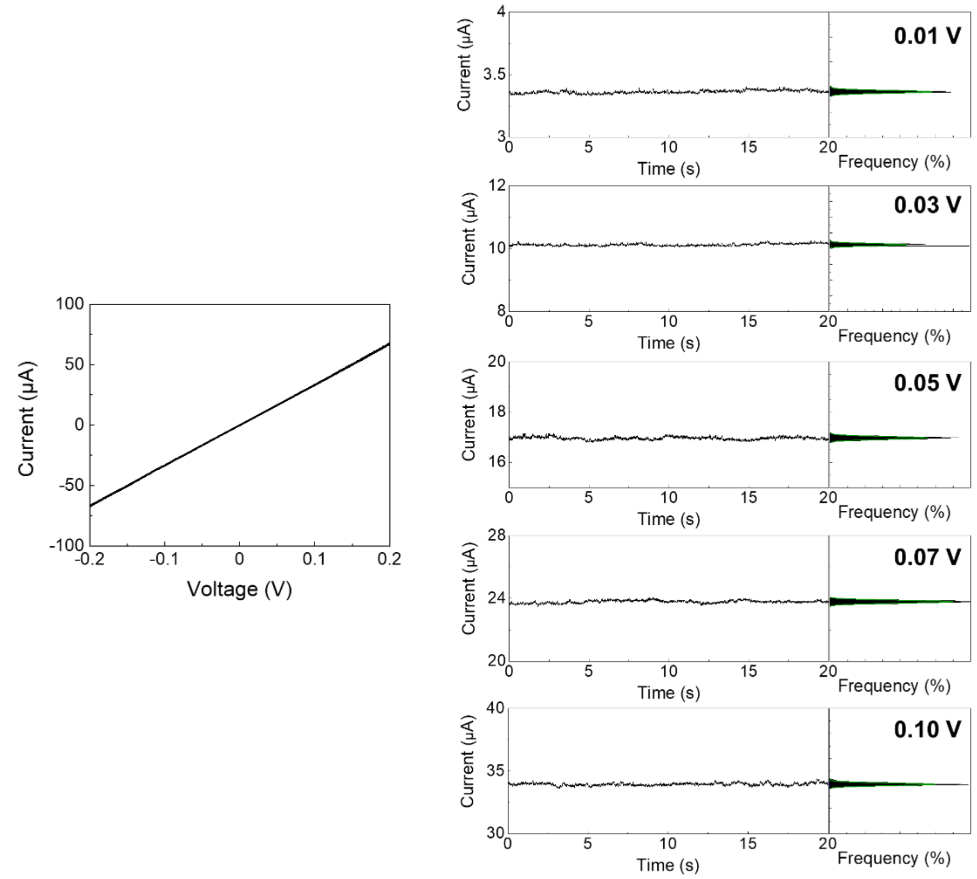

Figure S18: The current monitoring of the graphene homojunction device at different voltages. Left panel: *I*–*V* curve of the device of the graphene homojunction; Right panel: representative *I*–*t* trajectories and the corresponding histograms of the junction at 300 K and different voltages of 0.01 V, 0.03 V, 0.05 V, 0.07 V and 0.10 V.

Section 3: Theoretical calculation and analysis

Since the structures of the molecules are flexible, the initial configurations of the isolated molecules were generated by using a simulated annealing method in Gromacs program.[3] The molecular models were parameterized using a general amber force field (GAFF) with ACPYPE software.[4] During the annealing process, the peak temperature was set at 300 K, and the time length of each annealing cycle was set as 200 ps. Twenty annealing processes were implemented for each molecular model, and the configurations produced were further optimized, and the intrinsic molecular energy calculation for each configuration was carried out in the Gaussian 16 package[5] at B3LYP/6-31G(d) level, and the two configurations with lowest Gibbs free energy were chosen for further calculation.

The structural relaxation of the isolated molecules and two-probe transport junctions and the intrinsic molecular electronic structure calculation were carried out by the density functional theory (DFT) in the Gaussian 16 software package.[5] The B3LYP hybrid functional[6] was adopted in the theoretical calculation. The basis sets are employed as 6-31G(d) and 6-311G(d,p) for geometry optimization and electronic structure calculations, respectively. The charge transport properties of the two-probe structures were obtained by carrying out the density functional theory (DFT) within the nonequilibrium Green's function (NEGF) formalism, as implemented in the Atomistix Toolkit (ATK) package.[7–9] We adopted general gradient approximation (GGA) with a double-zeta plus polarization basis set. The density mesh cutoff was set as 125 Hartree. The NEGF-DFT self-consistent calculations were deemed converged when every element of the Hamiltonian matrix and the density matrix were converged to less than 10^{-5} a.u., and the total energy was converged to less than 10^{-5} a.u. A *k*-point mesh of 2×1×111 and 12×1 was used for device self-consistent calculations and conductance analysis (transmission eigenstate, transmission spectra and *I*–*V* curves) calculations, respectively.

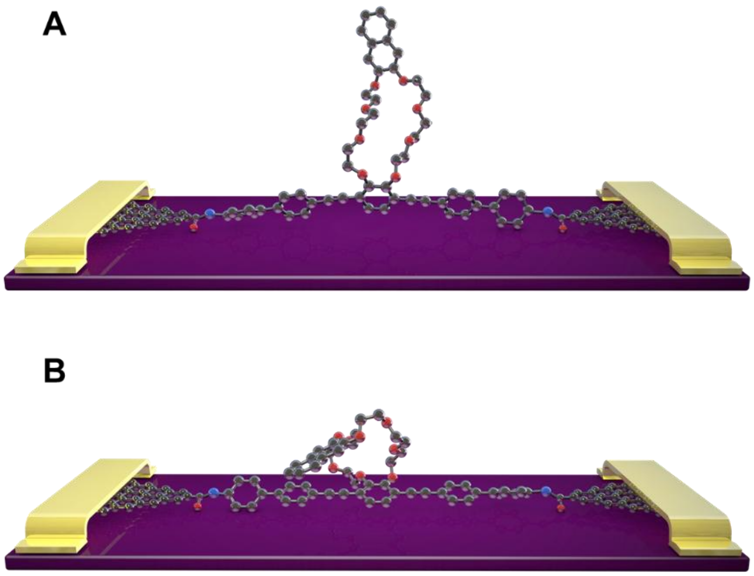

Figure S19: Schematic of the single-molecule junction at different structures. (**A**) Non-twisted molecular structure CE-I and (**B**) twisted molecular structure CE-II. The molecular structures are obtained through theoretical calculations.

Table S1: Energy difference between the two conformers calculated with the simulated annealing method.

Structure	CE-II	CE-I
Energy (Hatree)	-2875.541457	-2875.539010
Energy (kcal/mol)	0	1.54

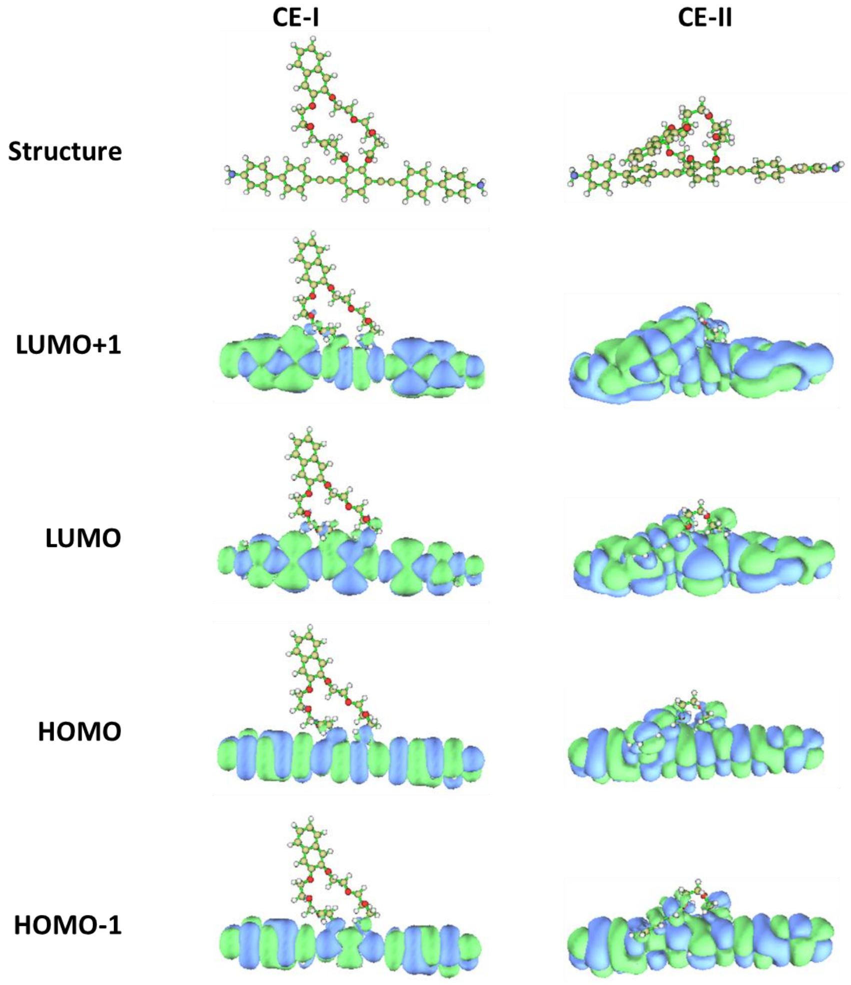

Figure S20: Molecular orbitals (MOs) of CE-I and CE-II. Molecular orbital diagrams of the two structures are exhibited (HOMO−1, HOMO, LUMO, and LUMO+1).

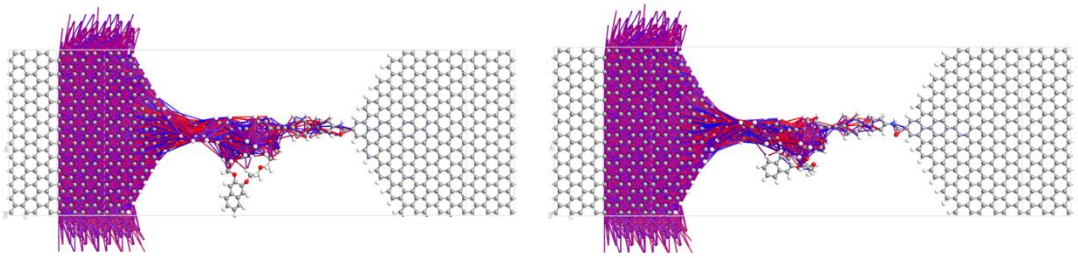

Figure S21: Local transmission path calculations of CE-I (left) and CE-II (right). Local transmission path from the main molecular bridge to the CE ring exist in the CE-II structure.

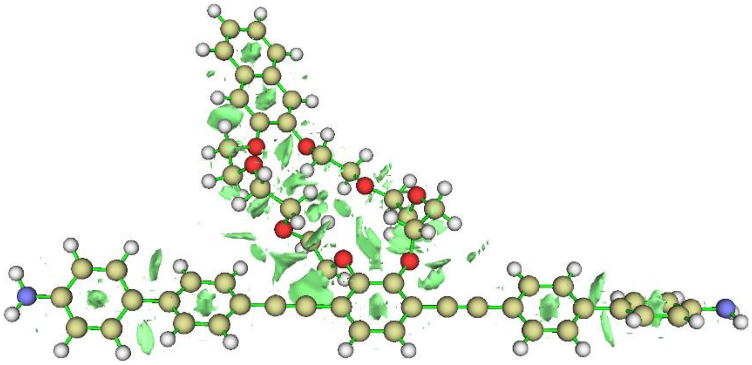

Figure S22: Non-covalent interaction analysis of CE-I, where green areas indicate the presence of weak interactions.

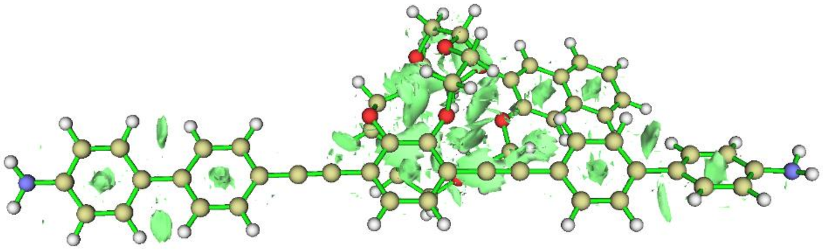

Figure S23: Non-covalent interaction analysis of CE-II, where green areas indicate the presence of weak interactions.

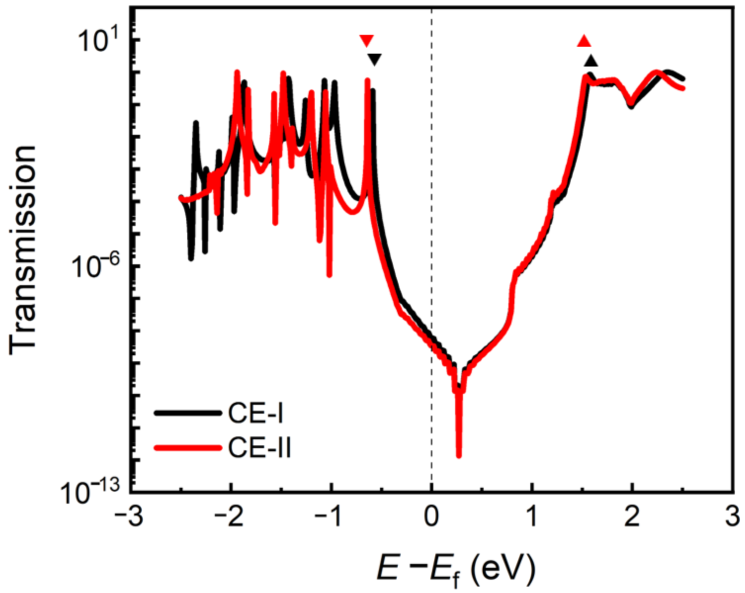

Figure S24: Calculated transmission spectra. Wide-range transmission spectra of the two structures of CE-I (black) and CE-II (red) indicate that the conductance of the single-molecule junction is dominated by perturbed highest occupied molecular orbitals (*p*-HOMOs). Triangles downwards denote *p*-HOMOs, while triangles upwards denote *p*-LUMOs.

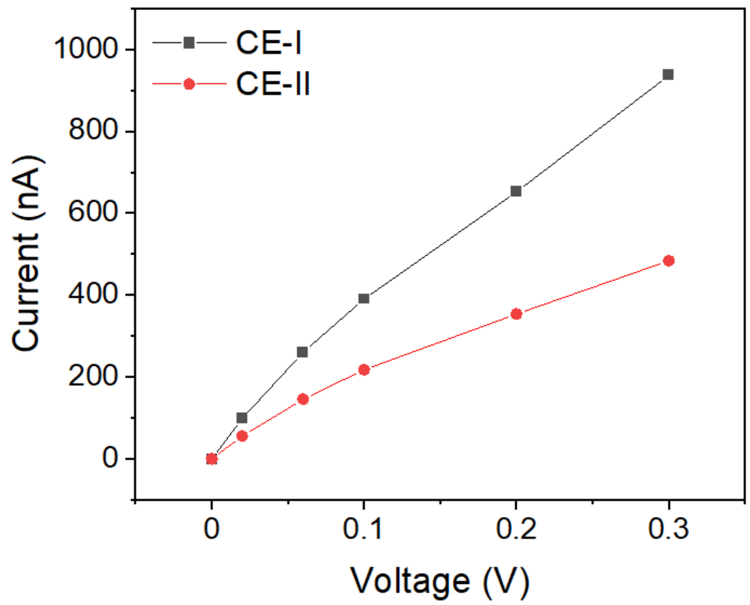

Figure S25: Calculated *I*–*V* curves. The *I*–*V* curves of the two structures of CE-I (black) and CE-II (red) are exhibited.

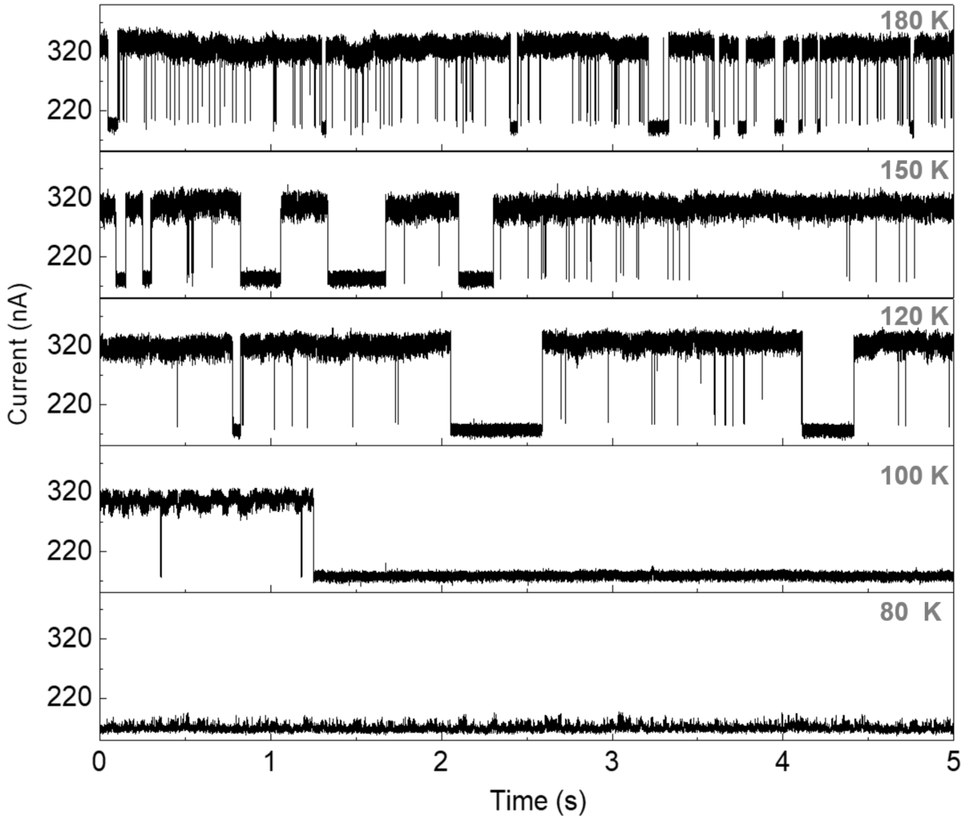

Figure S26: Detailed *I*–*t* curves of a single-molecule junction at low temperature (180 K~80 K).

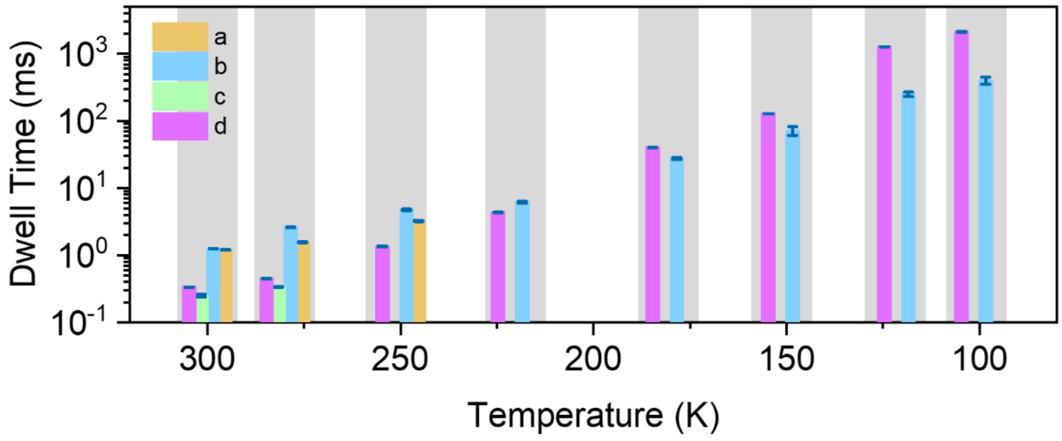

Figure S27: The dwell times of a single-molecule junction at 300 K~100 K, a, b, c and d denote CE-Ia, CE-I, CE-IIa and CE-II.

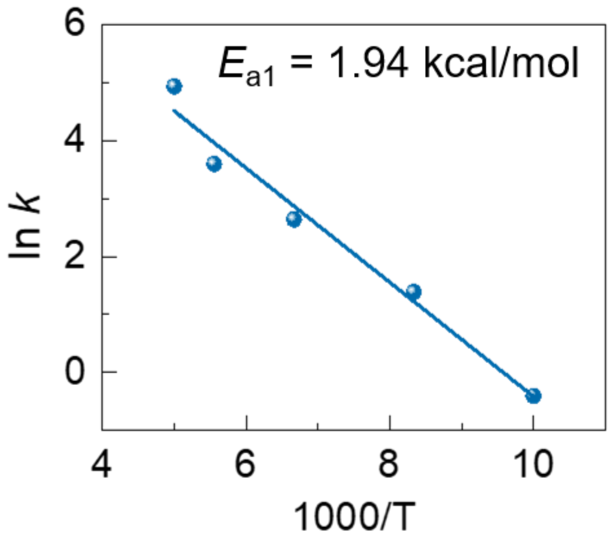

Figure S28: Plots of the transition rate (from CE-II to CE-I) deduced from temperature-dependent measurements.

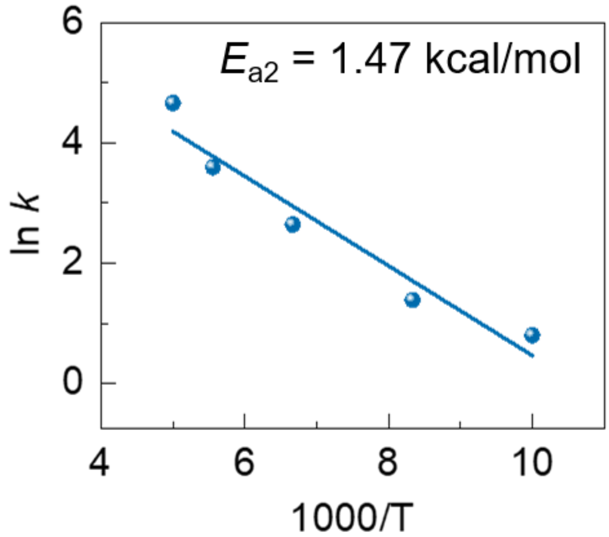

Figure S29: Plots of the transition rate (from CE-I to CE-II) deduced from temperature-dependent measurements.

Calculated interaction energy under external electric fields

The energy difference in the parallel and antiparallel configuration with the external electric field (EEF) was estimated based on the dipole moment in Figure 1D,E.

The coupling between molecular dipoles and the electric fields was calculated as the EEF-dipole interaction, where the Hamiltonian can be expressed as:

$$\hat{H}_E = -\int d^3r\, \hat{E}(r) \cdot \hat{P}(r)$$

where E(r) is the electric vector and P(r) is the molecular dipole moment. Assuming that the electric field is uniform, the EEF-dipole correlation energy E_{ED} can be obtained as follows:

$$E_{\mathrm{ED}} = E \cdot P$$

$$\rho_E \equiv \frac{E_{\mathrm{ED-I}}}{E_{\mathrm{ED-II}}} = \frac{E \cdot P_I}{E \cdot P_{II}} = \frac{0.97}{1.69} \approx 0.57$$

where E is the electric vector, P_{I} and P_{II} is the dipole moment of different molecular states, $E_{\mathrm{ED-I}}$ and $E_{\mathrm{ED-II}}$ denote the interaction between EEF and two states of molecule. And, ρ_{E} refers to the ratio of the interaction between these two states and EEF.[10]

The energy difference between CE-I and CE-II in the parallel and anti-parallel EEF are calculated as follows:

Table S2: Calculated energy difference between CE-I and CE-II in the parallel and anti-parallel EEF.

EEF-Dipole energy (a.u.)	Parallel EEF	Anti-parallel EEF
CE-I	0.57	–0.57
CE-II	1	−1

The system is more stable with more positive EEF-Dipole energy. Under the positive electric field, the increase of electric field will enrich CE-II. However, under the negative electric field, the increase of electric field will enrich CE-I, which is in good agreement with the experimental results in Figure 3C,D.

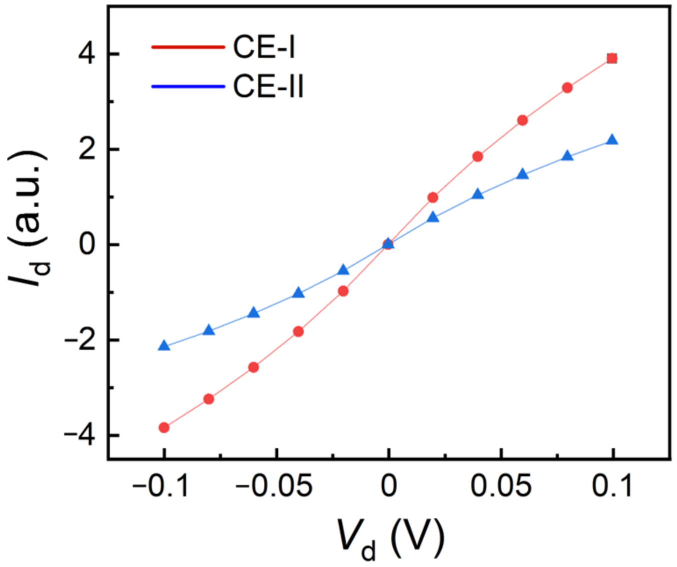

Figure S30: Calculated *I*–*V* curves of CE-I and CE-II. For the flat structure, we calculated the *I*–*V* curves under the voltage range of –0.1 V to 0.1 V, implemented in the QuantumATK package.

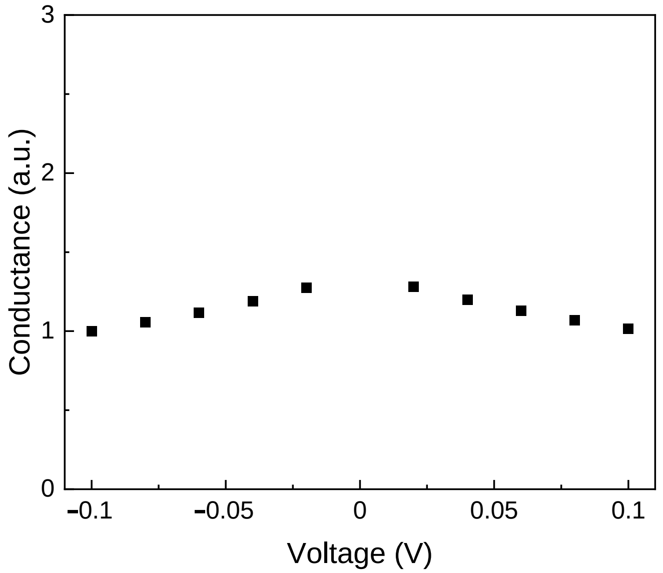

Figure S31: Calculated conductance changes of CE-II under different external electric voltages. For the calculation of the conductance graph shown in Figure S31, each conductance value was calculated as current value over the corresponding voltage value.

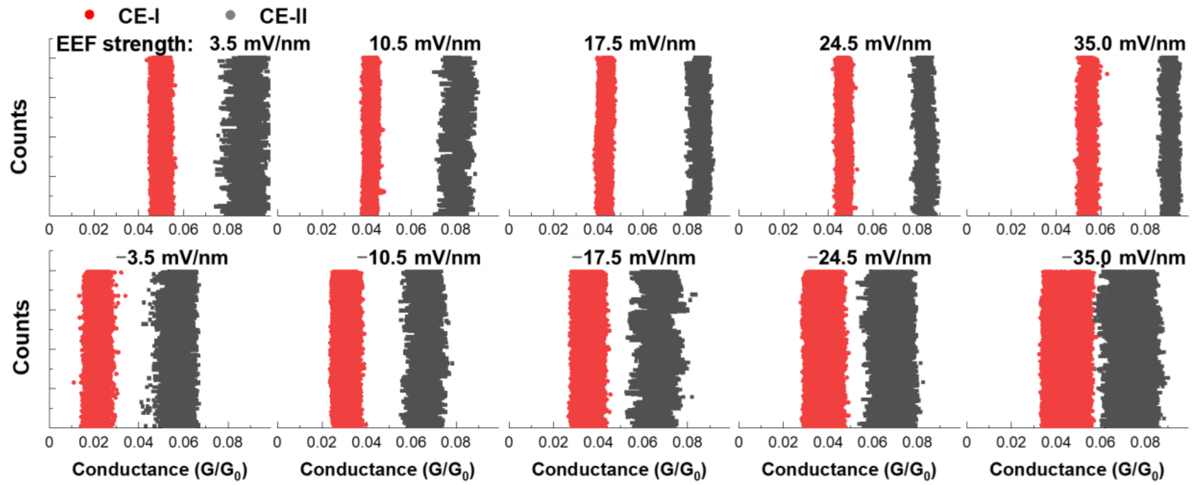

Figure S32: Asymmetric EEF responses of the conductance. The conductance distribution of CE-I and CE-II under various external electric field strengths of 3.5 mV/nm, 10.5 mV/nm, 17.5 mV/nm, 24.5 mV/nm, and 35.0 mV/nm, and the corresponding negative electric fields.

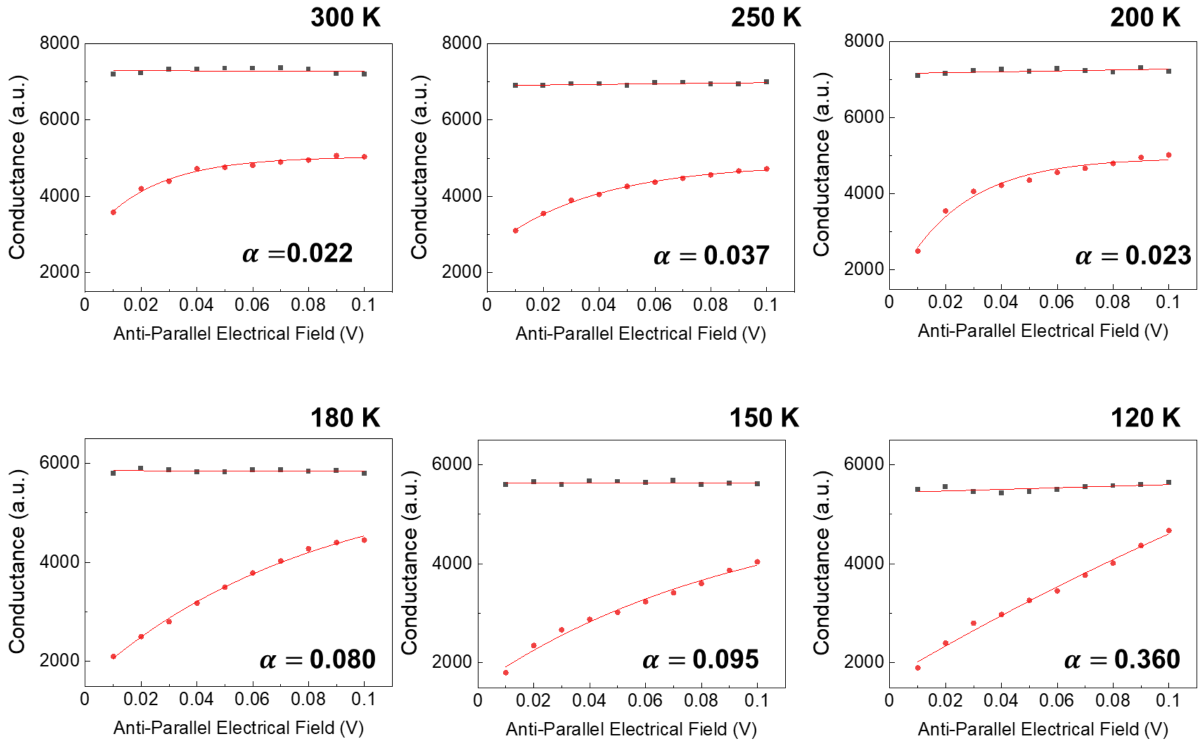

Figure S33: Conductance changes of the CE-I (high state) and CE-II (low state) under different temperatures.

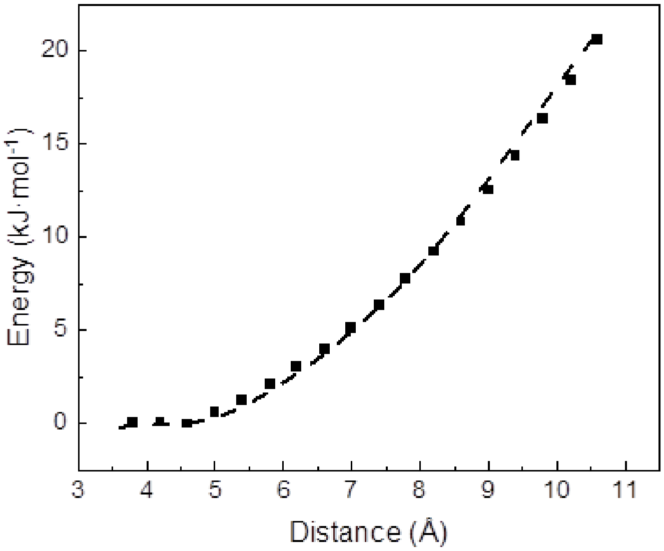

Figure S34: Single-point energy changes as the distance between the crown ether ring and the molecular bridge increases. Different conformations can be provided by changing the dihedral angle between the crown ether ring and the backbone.

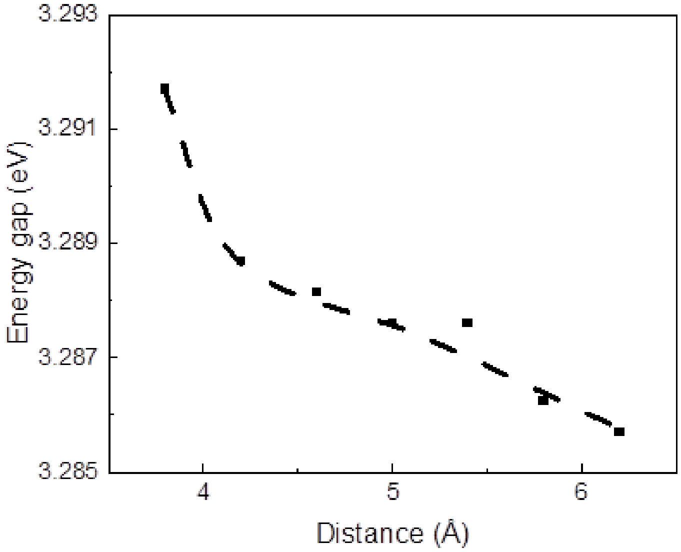

Figure S35: The HOMO/LUMO gap changes as the distance between the crown ether ring and the molecular bridge increases.

Table S3: Molecular orbital change as the distance between the crown ether ring and the molecular bridge increases.

d **(Å)**	**LUMO (eV)**	**HOMO (eV)**	**Gap (eV)**	**Dipole moment (Debye)**
3.8	–1.808	–5.100	3.291	2.188
4.2	–1.810	–5.099	3.288	2.121
4.6	–1.794	–5.082	3.288	2.135
5	–1.792	–5.079	3.287	2.111
5.4	–1.790	–5.078	3.287	2.088
5.8	–1.790	–5.076	3.286	2.067
6.2	–1.789	–5.075	3.285	2.041

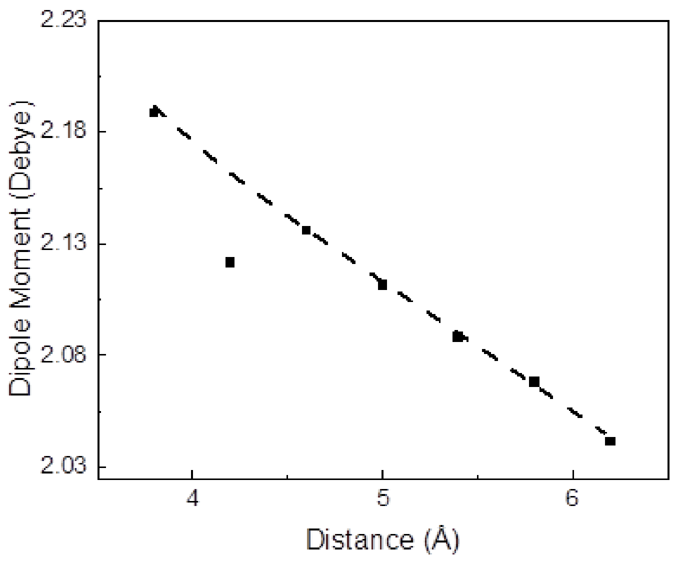

Figure S36: Dipole moment changes as the distance between the crown ether ring and the molecular bridge increases.

References

[1] S. Chen, D. Su, C. Jia, Y. Li, X. Li, X. Guo, D. A. Leigh, L. Zhang, *Chem* **2022**, *8*, 243.

[2] C. Yang, C. Yang, Y. Guo, J. Feng, X. Guo, *Nat. Protoc.* **2023**, *18*, 1958.

[3] M. J. Abraham, T. Murtola, R. Schulz, S. Páll, J. C. Smith, B. Hess, E. Lindahl, *Softwarex* **2015**, *1*, 19.

[4] A. W. Sousa da Silva, W. F. Vranken, *BMC Res. Notes* **2012**, *5*, 367.

[5] M. J. Frisch, G. W. Trucks, H. B. Schlegel, G. E. Scuseria, M. A. Robb, J. R. Cheeseman, G. Scalmani, V. Barone, G. A. Petersson, H. Nakatsuji, et al. *Gaussian 16 rev.* C.01. Wallingford, CT **2016**.

[6] A. D. Becke, *J. Chem. Phys.* **1993**, *98,* 5648.

[7] M. Brandbyge, J. -L. Mozos, P. Ordejón, J. Taylor, K. Stokbro, *Phys. Rev. B* **2002**, *65*, 165401.

[8] J. Taylor, H. Guo, J. Wang, *Phys. Rev.* B **2001**, *63*, 245407.

[9] S. Smidstrup, T. Markussen, P. Vancraeyveld, J. Wellendorff, J. Schneider, T. Gunst, B. Verstichel, D. Stradi, P. A. Khomyakov, U. G. Vej-Hansen, *J. Phys. Condens. Matter* **2019**, *32*, 015901.

[10] W. Hu, Z. Zhang, W. Xiong, M. Li, Y. Yan, C. Yang, Q. Zou, J.-T. Lü, H. Tian, X. Guo, *Sci. Adv.* **2024**, *10,* eado1125.
